# Supplementary material for: Prognostic 18F-FDG Radiomic Features in Advanced High-Grade Serous Ovarian Cancer
Source: Diagnostics (Basel). 2023 Nov 7;13(22):3394. doi: 10.3390/diagnostics13223394 (PMC10670627; doi:10.3390/diagnostics13223394)
Supplement: Supplementary file 1 [file diagnostics-13-03394-s001.zip › Radiomics statistic results 2.0.pdf]

DISEASE-FREE SURVIVAL

We look at the column of Sig. if the value is less than 0.05 that variable is significant and if it is greater or equal it is not.

Cox regression

Variables in the equation

	B	SE	Wald	gl	Sig.	Exp(B)	95.0% CI for Exp(B) Lower	95.0% CI for Exp(B) Top
MORPHOLOGICAL_ApproximateVolume	,000	,000	1,781	1	,182	1,000	1,000	1,000

Means of covariates

	Media
MORPHOLOGICAL_ApproximateVolume	1035211,182

Does not affect survival

Cox regression

Variables in the equation

	B	SE	Wald	gl	Sig.	Exp(B)	95.0% CI for Exp(B) Lower	95.0% CI for Exp(B) Top
MORPHOLOGICAL_Compacity	,190	,193	,967	1	,325	1,209	,828	1,766

Means of covariates

	Media
MORPHOLOGICAL_Compacity	2,396

Does not affect survival

Cox regression

Variables in the equation

	B	SE	Wald	gl	Sig.	Exp(B)	95.0% CI for Exp(B) Lower	Top
MORPHOLOGICAL_Compactness2	-3,538	3,459	1,047	1	,306	,029	,000	25,546

Means of covariates

	Media
MORPHOLOGICAL_Compactness2	,065

Does not affect survival

Cox regression

Variables in the equation

	B	SE	Wald	gl	Sig.	Exp(B)	95.0% CI for Exp(B) Lower	Top
MORPHOLOGICAL_CentreOfMassShift	,044	,031	1,954	1	,162	1,045	,983	1,111

Means of covariates

	Media
MORPHOLOGICAL_CentreOfMassShift	6,483

Does not affect survival

Cox regression

Variables in the equation

	B	SE	Wald	gl	Sig.	Exp(B)	95.0% CI for Exp(B) Lower	Top
INTENSITY-BASED_Variance	,004	,017	,059	1	,808	1,004	,971	1,039

Means of covariates

	Media
INTENSITY-BASED_Variance	8,107

Does not affect survival

Cox regression

Variables in the equation

	B	SE	Wald	gl	Sig.	Exp(B)	95.0% CI for Exp(B)	
							Lower	Top
INTENSITY-BASED_Kurtosis	,024	,020	1,485	1	,223	1,024	,985	1,065

Means of covariates

	Media
INTENSITY-BASED_Kurtosis	4,291

Does not affect survival

Cox regression

Variables in the equation

	B	SE	Wald	gl	Sig.	Exp(B)	95.0% CI for Exp(B)	
							Lower	Top
INTENSITY-BASED_MinimumGreyLevel	,150	,275	,297	1	,586	1,161	,678	1,989

Means of covariates

	Media
INTENSITY-BASED_MinimumGreyLevel	,851

Does not affect survival

Cox regression

Variables in the equation

	B	SE	Wald	gl	Sig.	Exp(B)	95.0% CI for Exp(B)	
							Lower	Top
INTENSITY-BASED_TotalLesionGlycolysis	,000	,000	4,168	1	,041	1,000	1,000	1,000

Means of covariates

	Media
INTENSITY-BASED_TotalLesionGlycolysis	3887,596

p=0.041 affects survival

Exp(B)=1.000130 => for each unit increase in the variable, the risk of relapse increases by 0.013%.

Cox regression

Variables in the equation

	B	SE	Wald	gl	Sig.	Exp(B)	95.0% CI for Exp(B)	
							Lower	Top
INTENSITY-HISTOGRAM_Uniformity	-,510	3,468	,022	1	,883	,601	,001	538,089

Means of covariates

	Media
INTENSITY-HISTOGRAM_Uniformity	,077

Does not affect survival

Cox regression

Variables in the equation

	B	SE	Wald	gl	Sig.	Exp(B)	95.0% CI for Exp(B)	
							Lower	Top
GLCM_JointMaximum	,360	2,871	,016	1	,900	1,433	,005	398,200

Means of covariates

	Media
GLCM_JointMaximum	,041

Does not affect survival

Cox regression

Variables in the equation

	B	SE	Wald	gl	Sig.	Exp(B)	95.0% CI for Exp(B)	
							Lower	Top
GLCM_InverseDifferenceMoment	,215	1,544	,019	1	,889	1,240	,060	25,575

Means of covariates

	Media
GLCM_InverseDifferenceMoment	,285

Does not affect survival

Cox regression

Variables in the equation

	B	SE	Wald	gl	Sig.	Exp(B)	95.0% CI for Exp(B)	
							Lower	Top
GLCM_InverseVariance	18,477	16,899	1,195	1	,274	105792585,0	,000	2,565E+22

Means of covariates

	Media
GLCM_InverseVariance	,017

Does not affect survival

Cox regression

Variables in the equation

	B	SE	Wald	gl	Sig.	Exp(B)	95.0% CI for Exp(B)	
							Lower	Top
GLCM_Correlation	-,412	1,582	,068	1	,794	,662	,030	14,703

Means of covariates

	Media
GLCM_Correlation	,670

Does not affect survival

Cox regression

	B	SE	Wald	gl	Sig.	Exp(B)	95.0% CI for Exp(B)	
							Lower	Top
GLCM_ClusterTendency	,000	,001	,008	1	,928	1,000	,998	1,002

Means of covariates

	Media
GLCM_ClusterTendency	231,505

Does not affect survival

Cox regression

Variables in the equation

	B	SE	Wald	gl	Sig.	Exp(B)	95.0% CI for Exp(B)	
							Lower	Top
GLCM_ClusterShade	,000	,000	,094	1	,759	1,000	1,000	1,000

Means of covariates

	Media
GLCM_ClusterShade	2692,499

Does not affect survival

Cox regression

Variables in the equation

	B	SE	Wald	gl	Sig.	Exp(B)	95.0% CI for Exp(B)	
							Lower	Top
GLRLM_LongRunsEmphasis	,058	,542	,011	1	,915	1,060	,366	3,064

Means of covariates

	Media
GLRLM_LongRunsEmphasis	1,432

Does not affect survival

Cox regression

	B	SE	Wald	gl	Sig.	Exp(B)	95.0% CI for Exp(B)	
							Lower	Top
GLRLM_RunLengthNonUniformity	,000	,000	3,528	1	,060	1,000	1,000	1,000

Means of covariates

	Media
GLRLM_RunLengthNonUniformity	11216,084

p=0.060 not significant but some association can be seen.

Cox regression

Variables in the equation

	B	SE	Wald	gl	Sig.	Exp(B)	95.0% CI for Exp(B)	
							Lower	Top
NGTDM_Coarseness	-15,237	46,952	,105	1	,746	,000	,000	2,229E+33

Means of covariates

	Media
NGTDM_Coarseness	,003

Does not affect survival

Cox regression

Variables in the equation

	B	SE	Wald	gl	Sig.	Exp(B)	95.0% CI for Exp(B)	
							Lower	Top
NGTDM_Busyness	,003	,059	,002	1	,962	1,003	,893	1,126

Means of covariates

	Media
NGTDM_Busyness	3,152

Does not affect survival

Cox regression

	B	SE	Wald	gl	Sig.	Exp(B)	95.0% CI for Exp(B)	
							Lower	Top
NGTDM_Complexity	,000	,000	,032	1	,858	1,000	1,000	1,000

Means of covariates

	Media
NGTDM_Complexity	3013,886

Does not affect survival

Cox regression

Variables in the equation

	B	SE	Wald	gl	Sig.	Exp(B)	95.0% CI for Exp(B)	
							Lower	Top
GLSZM_LargeZoneHighGreyLevelEmphasis	,000	,000	1,005	1	,316	1,000	1,000	1,000

Means of covariates

	Media
GLSZM_LargeZoneHighGreyLevelEmphasis	250301,023

Does not affect survival

Cox regression

Variables in the equation

	B	SE	Wald	gl	Sig.	Exp(B)	95.0% CI for Exp(B)	
							Lower	Top
GLSZM_ZoneSizeNonUniformity	,000	,000	4,719	1	,030	1,000	1,000	1,001

Means of covariates

	Media
GLSZM_ZoneSizeNonUniformity	1301,301

p=0.030 affects survival
Exp(B)=1.000328 => for each unit increase in the variable, the risk of relapse increases by 0.0328%.

Cox regression

Variables in the equation

	B	SE	Wald	gl	Sig.	Exp(B)	95.0% CI for Exp(B) Lower	Top
GLSZM_NormalisedZoneSizeNonUniformity	-,230	1,817	,016	1	,899	,794	,023	27,959

Means of covariates

	Media
GLSZM_NormalisedZoneSizeNonUniformity	,380

```
Does not affect survival
```

OVERALL SURVIVAL

Cox regression

Variables in the equation

	B	SE	Wald	gl	Sig.	Exp(B)	95.0% CI for Exp(B) Lower	Top
MORPHOLOGICAL_ApproximateVolume	,000	,000	,139	1	,710	1,000	1,000	1,000

Means of covariates

	Media
MORPHOLOGICAL_ApproximateVolume	1025659,879

```
Does not affect survival
```

Cox regression

Variables in the equation

	B	SE	Wald	gl	Sig.	Exp(B)	95.0% CI for Exp(B) Lower	Top
MORPHOLOGICAL_Compacity	-,458	,351	1,699	1	,192	,633	,318	1,260

Means of covariates

	Media
MORPHOLOGICAL_Compacity	2,372

```
Does not affect survival
```

Cox regression

Variables in the equation

	B	SE	Wald	gl	Sig.	Exp(B)	95.0% CI for Exp(B)	
							Lower	Top
MORPHOLOGICAL_Compactness2	-3,930	5,158	,581	1	,446	,020	,000	482,884

Means of covariates

	Media
MORPHOLOGICAL_Compactness2	,064

```
Does not affect survival
```

Cox regression

Variables in the equation

	B	SE	Wald	gl	Sig.	Exp(B)	95.0% CI for Exp(B)	
							Lower	Top
MORPHOLOGICAL_CentreOfMassShift	,018	,034	,295	1	,587	1,019	,953	1,089

Means of covariates

	Media
MORPHOLOGICAL_CentreOfMassShift	6,367

```
Does not affect survival
```

Cox regression

Variables in the equation

	B	SE	Wald	gl	Sig.	Exp(B)	95.0% CI for Exp(B)	
							Lower	Top
INTENSITY-BASED_Variance	-,001	,024	,003	1	,960	,999	,952	1,047

Means of covariates

	Media
INTENSITY-BASED_Variance	7,899

Does not affect survival

Cox regression

Variables in the equation

	B	SE	Wald	gl	Sig.	Exp(B)	95.0% CI for Exp(B)	
							Lower	Top
INTENSITY-BASED_Kurtosis	,047	,021	4,867	1	,027	1,048	1,005	1,093

Means of covariates

	Media
INTENSITY-BASED_Kurtosis	4,281

p=0.027 affects survival

Exp(B)=1.048 => for each unit increase in the variable, the risk of exitus increases by 4.8%.

Cox regression

Variables in the equation

	B	SE	Wald	gl	Sig.	Exp(B)	95.0% CI for Exp(B)	
							Lower	Top
INTENSITY-BASED_MinimumGreyLevel	,259	,355	,534	1	,465	1,296	,647	2,596

Means of covariates

	Media
INTENSITY-BASED_MinimumGreyLevel	,853

Does not affect survival

Cox regression

Variables in the equation

	B	SE	Wald	gl	Sig.	Exp(B)	95.0% CI for Exp(B)	
							Lower	Top
INTENSITY-BASED_TotalLesionGlycolysis	,000	,000	,113	1	,737	1,000	1,000	1,000

Means of covariates

	Media
INTENSITY-BASED_TotalLesionGlycolysis	3828,082

Does not affect survival

Cox regression

Variables in the equation

	B	SE	Wald	gl	Sig.	Exp(B)	95.0% CI for Exp(B)	
							Lower	Top
INTENSITY-HISTOGRAM_Uniformity	5,760	4,048	2,024	1	,155	317,396	,114	886410,530

Means of covariates

	Media
INTENSITY-HISTOGRAM_Uniformity	,079

Does not affect survival

Cox regression

Variables in the equation

	B	SE	Wald	gl	Sig.	Exp(B)	95.0% CI for Exp(B)	
							Lower	Top
GLCM_JointMaximum	3,465	2,597	1,781	1	,182	31,984	,197	5190,139

Means of covariates

	Media
GLCM_JointMaximum	,041

Does not affect survival

Cox regression

Variables in the equation

	B	SE	Wald	gl	Sig.	Exp(B)	95.0% CI for Exp(B)	
							Lower	Top
GLCM_InverseDifferenceMoment	3,342	2,125	2,474	1	,116	28,268	,439	1819,217

Means of covariates

	Media
GLCM_InverseDifferenceMoment	,289

Does not affect survival

Cox regression

Variables in the equation

	B	SE	Wald	gl	Sig.	Exp(B)	95.0% CI for Exp(B)	
							Lower	Top
GLCM_InverseVariance	12,887	19,948	,417	1	,518	395229,287	,000	3,770E+22

Means of covariates

	Media
GLCM_InverseVariance	,017

Does not affect survival

Cox regression

Variables in the equation

	B	SE	Wald	gl	Sig.	Exp(B)	95.0% CI for Exp(B)	
							Lower	Top
GLCM_Correlation	-,924	2,001	,213	1	,644	,397	,008	20,051

Means of covariates

	Media
GLCM_Correlation	,667

```
Does not affect survival
```

Cox regression

Variables in the equation

	B	SE	Wald	gl	Sig.	Exp(B)	95.0% CI for Exp(B)	
							Lower	Top
GLCM_ClusterTendency	-,003	,002	1,621	1	,203	,997	,993	1,001

Means of covariates

	Media
GLCM_ClusterTendency	225,731

```
Does not affect survival
```

Cox regression

Variables in the equation

	B	SE	Wald	gl	Sig.	Exp(B)	95.0% CI for Exp(B)	
							Lower	Top
GLCM_ClusterShade	,000	,000	1,125	1	,289	1,000	1,000	1,000

Means of covariates

	Media
GLCM_ClusterShade	2622,213

```
Does not affect survival
```

Cox regression

Variables in the equation

	B	SE	Wald	gl	Sig.	Exp(B)	95.0% CI for Exp(B)	
							Lower	Top
GLRLM_LongRunsEmphasis	,828	,548	2,277	1	,131	2,288	,781	6,702

Means of covariates

	Media
GLRLM_LongRunsEmphasis	1,435

Does not affect survival

Cox regression

Variables in the equation

	B	SE	Wald	gl	Sig.	Exp(B)	95.0% CI for Exp(B)	
							Lower	Top
GLRLM_RunLengthNonUniformity	,000	,000	,082	1	,775	1,000	1,000	1,000

Means of covariates

	Media
GLRLM_RunLengthNonUniformity	11100,407

Does not affect survival

Cox regression

Variables in the equation

	B	SE	Wald	gl	Sig.	Exp(B)	95.0% CI for Exp(B)	
							Lower	Top
NGTDM_Coarseness	-62,888	86,289	,531	1	,466	,000	,000	1,372E+46

Means of covariates

	Media
NGTDM_Coarseness	,003

Does not affect survival

Cox regression

Variables in the equation

	B	SE	Wald	gl	Sig.	Exp(B)	95.0% CI for Exp(B)	
							Lower	Top
NGTDM_Busyness	,053	,079	,450	1	,502	1,055	,903	1,233

Means of covariates

	Media
NGTDM_Busyness	3,205

Does not affect survival

Cox regression

Variables in the equation

	B	SE	Wald	gl	Sig.	Exp(B)	95.0% CI for Exp(B)	
							Lower	Top
NGTDM_Complexity	,000	,000	,036	1	,849	1,000	1,000	1,000

Means of covariates

	Media
NGTDM_Complexity	2940,465

Does not affect survival

Cox regression

Variables in the equation

	B	SE	Wald	gl	Sig.	Exp(B)	95.0% CI for Exp(B)	
							Lower	Top
GLSZM_LargeZoneHighGreyLevelEmphasis	,000	,000	,001	1	,974	1,000	1,000	1,000

Means of covariates

	Media
GLSZM_LargeZoneHighGreyLevelEmphasis	250689,323

Does not affect survival

Cox regression

Variables in the equation

	B	SE	Wald	gl	Sig.	Exp(B)	95.0% CI for Exp(B)	
							Lower	Top
GLSZM_ZoneSizeNonUniformity	,000	,000	,909	1	,340	1,000	,999	1,000

Means of covariates

	Media
GLSZM_ZoneSizeNonUniformity	1275,701

Does not affect survival

Cox regression

Variables in the equation

	B	SE	Wald	gl	Sig.	Exp(B)	95.0% CI for Exp(B)	
							Lower	Top
GLSZM_NormalisedZoneSizeNonUniformity	-,676	2,734	,061	1	,805	,509	,002	108,112

Means of covariates

	Media
GLSZM_NormalisedZoneSizeNonUniformity	,377

Does not affect survival

RADIOMICS FEATURES WITH GRAY LEVELS RELATIVE RESAMPLING

DISEASE-FREE SURVIVAL

We look at the column of Sig. if the value is less than 0.05 that variable is significant and if it is greater or equal it is not.

Cox regression

Variables in the equation

	B	SE	Wald	gl	Sig.	Exp(B)	95.0% CI for Exp(B)	
							Lower	Top
GLCM_AngularSecondMoment	-6,354	7,976	,635	1	,426	,002	,000	10710,180

Means of covariates

	Media
GLCM_AngularSecondMoment	,020

Does not affect survival

Cox regression

Variables in the equation

	B	SE	Wald	gl	Sig.	Exp(B)	95.0% CI for Exp(B)	
							Lower	Top
GLCM_InverseVariance	10,589	15,450	,470	1	,493	39703,748	,000	5,619E+17

Means of covariates

	Media
GLCM_InverseVariance	,060

Does not affect survival

Cox regression

Variables in the equation

	B	SE	Wald	gl	Sig.	Exp(B)	95.0% CI for Exp(B)	
							Lower	Top
GLCM_Correlation	-,435	1,617	,072	1	,788	,647	,027	15,389

Means of covariates

	Media
GLCM_Correlation	,668

Does not affect survival

Cox regression

Variables in the equation

	B	SE	Wald	gl	Sig.	Exp(B)	95.0% CI for Exp(B)	
							Lower	Top
GLCM_ClusterShade	,000	,000	,332	1	,564	1,000	1,000	1,000

Means of covariates

	Media
GLCM_ClusterShade	1019,322

Does not affect survival

Cox regression

Variables in the equation

	B	SE	Wald	gl	Sig.	Exp(B)	95.0% CI for Exp(B)	
							Lower	Top
GLRLM_ShortRunLowGreyLevelEmphasis	-2,354	8,030	,086	1	,769	,095	,000	650667,433

Means of covariates

	Media
GLRLM_ShortRunLowGreyLevelEmphasis	,048

Does not affect survival

Cox regression

Variables in the equation

	B	SE	Wald	gl	Sig.	Exp(B)	95.0% CI for Exp(B)	
							Lower	Top
GLRLM_ShortRunHighGreyLevelEmphasis	,000	,001	,068	1	,794	1,000	,998	1,002

Means of covariates

	Media
GLRLM_ShortRunHighGreyLevelEmphasis	138,908

Does not affect survival

Cox regression

Variables in the equation

	B	SE	Wald	gl	Sig.	Exp(B)	95.0% CI for Exp(B)	
							Lower	Top
GLRLM_RunLengthNonUniformity	,000	,000	4,005	1	,045	1,000	1,000	1,000

Means of covariates

	Media
GLRLM_RunLengthNonUniformity	9429,478

p=0.045 this variable affects survival.

Exp(B)=1.000057 => For each unit increase in this variable the risk of relapse increases by 0.0057%.

Cox regression

Variables in the equation

	B	SE	Wald	gl	Sig.	Exp(B)	95.0% CI for Exp(B)	
							Lower	Top
NGTDM_Coarseness	-3,295	39,178	,007	1	,933	,037	,000	8,269E+31

Means of covariates

	Media
NGTDM_Coarseness	,003

Does not affect survival

Cox regression

Variables in the equation

	B	SE	Wald	gl	Sig.	Exp(B)	95.0% CI for Exp(B) Lower	Top
NGTDM_Busyness	-,004	,021	,042	1	,838	,996	,955	1,038

Means of covariates

	Media
NGTDM_Busyness	8,415

Does not affect survival

Cox regression

Variables in the equation

	B	SE	Wald	gl	Sig.	Exp(B)	95.0% CI for Exp(B) Lower	Top
NGTDM_Complexity	,000	,000	,404	1	,525	1,000	1,000	1,000

Means of covariates

	Media
NGTDM_Complexity	1566,375

Does not affect survival

Cox regression

Variables in the equation

	B	SE	Wald	gl	Sig.	Exp(B)	95.0% CI for Exp(B) Lower	Top
GLSZM_SmallZoneEmphasis	,936	2,588	,131	1	,718	2,549	,016	406,479

Means of covariates

	Media
GLSZM_SmallZoneEmphasis	,579

Does not affect survival

Cox regression

Variables in the equation

	B	SE	Wald	gl	Sig.	Exp(B)	95.0% CI for Exp(B)	
							Lower	Top
GLSZM_LargeZoneEmphasis	,000	,000	,984	1	,321	1,000	1,000	1,000

Means of covariates

	Media
GLSZM_LargeZoneEmphasis	20166,220

Does not affect survival

Cox regression

Variables in the equation

	B	SE	Wald	gl	Sig.	Exp(B)	95.0% CI for Exp(B)	
							Lower	Top
GLSZM_SmallZoneLowGreandLevelEmphasis	-1,290	5,587	,053	1	,817	,275	,000	15696,991

Means of covariates

	Media
GLSZM_SmallZoneLowGreandLevelEmphasis	,035

Does not affect survival

Cox regression

Variables in the equation

	B	SE	Wald	gl	Sig.	Exp(B)	95.0% CI for Exp(B)	
							Lower	Top
GLSZM_GreyLevelNonUniformity	,005	,003	3,224	1	,073	1,005	1,000	1,011

Means of covariates

	Media
GLSZM_GreyLevelNonUniformity	111,156

p=0.073 some association is appreciated although the standards are not reached.

of significance.

OVERALL SURVIVAL

Cox regression

Variables in the equation

	B	SE	Wald	gl	Sig.	Exp(B)	95.0% CI for Exp(B)	
							Lower	Top
GLCM_AngularSecondMoment	4,469	9,291	,231	1	,631	87,251	,000	7071471617

Means of covariates

	Media
GLCM_AngularSecondMoment	,021

Does not affect survival

Cox regression

Variables in the equation

	B	SE	Wald	gl	Sig.	Exp(B)	95.0% CI for Exp(B)	
							Lower	Top
GLCM_InverseVariance	-14,961	17,645	,719	1	,396	,000	,000	332499211,9

Means of covariates

	Media
GLCM_InverseVariance	,060

Does not affect survival

Cox regression

Variables in the equation

	B	SE	Wald	gl	Sig.	Exp(B)	95.0% CI for Exp(B)	
							Lower	Top
GLCM_Correlation	-1,164	2,103	,306	1	,580	,312	,005	19,242

Means of covariates

	Media
GLCM_Correlation	,673

```
Does not affect survival
```

Cox regression

Variables in the equation

	B	SE	Wald	gl	Sig.	Exp(B)	95.0% CI for Exp(B)	
							Lower	Top
GLCM_ClusterShade	,000	,000	,622	1	,430	1,000	,999	1,000

Means of covariates

	Media
GLCM_ClusterShade	1021,148

```
Does not affect survival
```

Cox regression

Variables in the equation

	B	SE	Wald	gl	Sig.	Exp(B)	95.0% CI for Exp(B)	
							Lower	Top
GLRLM_ShortRunLowGrey LevelEmphasis	13,485	10,572	1,627	1	,202	718588,910	,001	7,164E+14

Means of covariates

	Media
GLRLM_ShortRunLowGrey LevelEmphasis	,050

```
Does not affect survival
```

Cox regression

Variables in the equation

	B	SE	Wald	gl	Sig.	Exp(B)	95.0% CI for Exp(B)	
							Lower	Top
GLRLM_ShortRunHighGreyLevelEmphasis	,000	,001	,087	1	,768	1,000	,998	1,003

Means of covariates

	Media
GLRLM_ShortRunHighGreyLevelEmphasis	136,484

Does not affect survival

Cox regression

Variables in the equation

	B	SE	Wald	gl	Sig.	Exp(B)	95.0% CI for Exp(B)	
							Lower	Top
GLRLM_RunLengthNonUniformity	,000	,000	,045	1	,832	1,000	1,000	1,000

Means of covariates

	Media
GLRLM_RunLengthNonUniformity	9585,530

Does not affect survival

Cox regression

Variables in the equation

	B	SE	Wald	gl	Sig.	Exp(B)	95.0% CI for Exp(B)	
							Lower	Top
NGTDM_Coarseness	-,837	56,537	,000	1	,988	,433	,000	5,767E+47

Means of covariates

	Media
NGTDM_Coarseness	,003

Does not affect survival

Cox regression

Variables in the equation

	B	SE	Wald	gl	Sig.	Exp(B)	95.0% CI for Exp(B)	
							Lower	Top
NGTDM_Busyness	,022	,027	,662	1	,416	1,023	,969	1,079

Means of covariates

	Media
NGTDM_Busyness	8,892

Does not affect survival

Cox regression

Variables in the equation

	B	SE	Wald	gl	Sig.	Exp(B)	95.0% CI for Exp(B)	
							Lower	Top
NGTDM_Complexity	,000	,000	,007	1	,931	1,000	1,000	1,000

Means of covariates

	Media
NGTDM_Complexity	1562,378

Cox regression

Variables in the equation

	B	SE	Wald	gl	Sig.	Exp(B)	95.0% CI for Exp(B)	
							Lower	Top
GLSZM_SmallZoneEmphasis	,376	3,836	,010	1	,922	1,456	,001	2683,163

Means of covariates

	Media
GLSZM_SmallZoneEmphasis	,578

Does not affect survival

Cox regression

Variables in the equation

	B	SE	Wald	gl	Sig.	Exp(B)	95.0% CI for Exp(B)	
							Lower	Top
GLSZM_LargeZoneEmphasis	,000	,000	,004	1	,952	1,000	1,000	1,000

Means of covariates

	Media
GLSZM_LargeZoneEmphasis	20809,502

Does not affect survival

Cox regression

Variables in the equation

	B	SE	Wald	gl	Sig.	Exp(B)	95.0% CI for Exp(B)	
							Lower	Top
GLSZM_SmallZoneLowGreandLevelEmphasis	7,439	6,688	1,237	1	,266	1700,446	,003	837850851,7

Means of covariates

	Media
GLSZM_SmallZoneLowGreandLevelEmphasis	,038

Does not affect survival

Cox regression

Variables in the equation

	B	SE	Wald	gl	Sig.	Exp(B)	95.0% CI for Exp(B)	
							Lower	Top
GLSZM_GreyLevelNonUniformity	-,001	,003	,133	1	,715	,999	,992	1,005

Means of covariates

	Media
GLSZM_GreyLevelNonUniformity	113,712

Does not affect survival

ROC RADIOMICS

1) DISEASE-FREE SURVIVAL

- Cut-off points for INTENSITY_BASED_TotalLesionsGlycolysis ,_ GLRLM_RunLengthNonUniformity and GLSZM_ZoneSiteNonUniformity according to relapse

COR Curve

Case Processing Summary

Relapse	N valid (per list)
Positive to Negative	26

Larger values of test result variables indicate a higher test for a positive true state.

a. The actual positive state is 1.

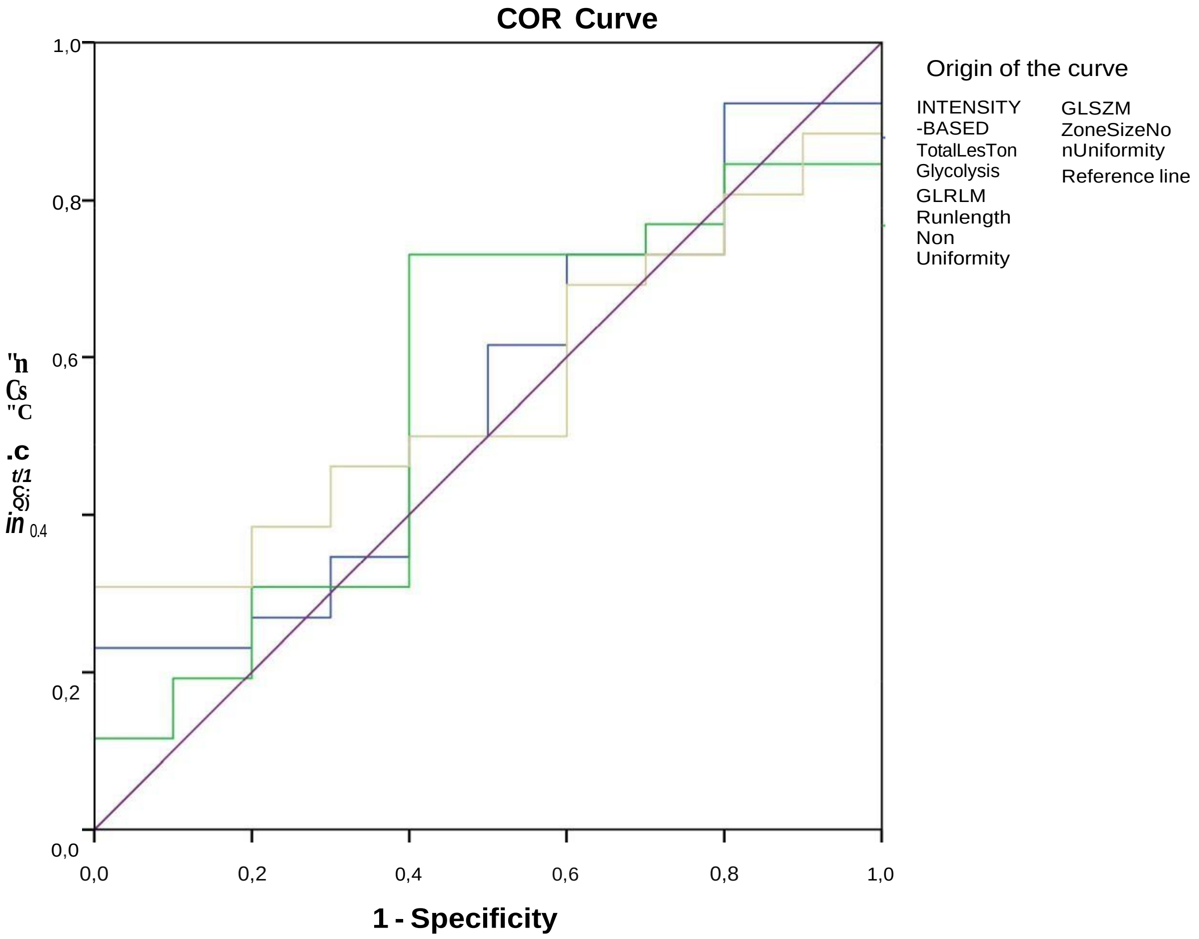

Area under the curve

Test result variables	Area	Standard Error[a]	Significance asint6ticab	95% confidence interval asymptotic	
				Lower limit	Upper limit
INTENSITY-BASED_TotallesionGlycolysis	,550	,105	,646	,345	,755
GLRLM_RunlengthNonUniformity	,558	,108	,596	,346	,769
GLSZM_ZoneSizeNon Uniformity	,558	,097	,596	,368	,747

a. Lower the non-parametric assumption

b. Null hypothesis: true area = 0,5

Curve coordinates

Test result variables	Positive if it is greater than or equal to	Sensitivity	1 - Specificity
INTENSITY-BASED_TotalLesionGlycolysis	152,9684044	1,000	1,000
	172,3748718	,962	1,000
	234,2939559	,923	1,000
	309,2503345	,923	,900
	374,3489828	,923	,800
	427,3920432	,885	,800
	477,1271677	,846	,800
	590,4759773	,808	,800
	761,5364248	,769	,800
	882,5457381	,731	,800
	1330,295876	,731	,700
	1868,916027	,731	,600
	2181,803669	,692	,600
	2413,333026	,654	,600
	2475,945106	,615	,600
	2512,790457	,615	,500
	2568,459607	,577	,500
	2708,755278	,538	,500
	2885,491495	,500	,500
	2974,573315	,500	,400
	3031,522482	,462	,400
	3487,141911	,423	,400
	3926,293697	,385	,400
	4095,310569	,346	,400
	4287,377258	,346	,300
	4765,109443	,308	,300
	5416,574117	,269	,300
	5642,196165	,269	,200
	5801,624457	,231	,200
	6043,847574	,231	,100
	6805,340681	,231	,000
	7765,084055	,192	,000
	9072,876245	,154	,000
	10245,05878	,115	,000
	10686,07768	,077	,000
	12001,31840	,038	,000
	13024,00172	,000	,000
GLRLM_RunLengthNonUniformity	193,8915964	1,000	1,000
	234,2044177	,962	1,000
	326,5781671	,923	1,000
	484,7107520	,885	1,000
	597,7216343	,846	1,000
	702,3783474	,846	,900
	1194,316272	,846	,800
	1626,943242	,808	,800
	2015,703130	,769	,800
	2407,469699	,769	,700

Curve coordinates

Test result variables	Positive if it is greater than or equal to	Sensitivity	1 - Specificity
GLSZM_ZoneSizeNonUniformity	4213,514197	,731	,700
	6515,430111	,731	,600
	7206,736869	,731	,500
	7388,320544	,731	,400
	7418,851743	,692	,400
	7602,663358	,654	,400
	7820,145921	,615	,400
	7866,261719	,577	,400
	8017,584584	,538	,400
	8441,839422	,500	,400
	8837,898349	,462	,400
	9234,779214	,423	,400
	9540,272414	,385	,400
	12157,16150	,346	,400
	14812,43157	,308	,400
	14880,70722	,308	,300
	14984,58743	,308	,200
	15895,67436	,269	,200
	17166,87422	,231	,200
	17751,55936	,192	,200
	18801,07858	,192	,100
	21676,01761	,154	,100
	24116,97121	,115	,100
	26263,53871	,115	,000
	31278,87177	,077	,000
	38677,66806	,038	,000
	42720,48754	,000	,000
	59,04721030	1,000	1,000
	72,96397212	,962	1,000
	101,9686689	,923	1,000
	140,4575472	,885	1,000
	168,9686542	,885	,900
	209,4484998	,846	,900
	253,7721344	,808	,900
	292,6998359	,808	,800
	325,5208415	,769	,800
	354,5436659	,731	,800
	396,7046220	,731	,700
	439,9362583	,692	,700
	493,3319939	,692	,600
	621,7938903	,654	,600
	824,8080576	,615	,600
	942,1356071	,577	,600
	976,4553701	,538	,600
	1018,014683	,500	,600
	1059,165687	,500	,500
	1103,973706	,500	,400

Curve coordinates

Test result variables	Positive if it is greater than or equal to	Sensitivity	1 - Specificity
	1133,141121	,462	,400
	1146,656329	,462	,300
	1192,479691	,423	,300
	1264,689992	,385	,300
	1319,053707	,385	,200
	1361,890823	,346	,200
	1545,004886	,308	,200
	1722,768296	,308	,100
	1803,478467	,308	,000
	1967,481586	,269	,000
	2219,631244	,231	,000
	2430,202628	,192	,000
	2512,318297	,154	,000
	2711,606325	,115	,000
	3974,310275	,077	,000
	5161,355791	,038	,000
	5265,964228	,000	,000

a. The smallest cutoff value is the minimum observed test value minus 1 and the largest cutoff value is the maximum observed test value plus 1. All other cutoff values are the averages of the two consecutive requested observed test values.

INTENSITY_BASED_TotalLesionGlycolys: cut-off point 2512.790457 which has a sensitivity of 61.5% and a specificity of 50%.

GLRLM_RunLengthNonUniformit:y cut-off point 7388.320544 which has a sensitivity of 73.1% and specificity of 60%.

GLSZM_ZoneSizeNonUniformit:and cutoff point 1103.973706 which has sensitivity of 50% and specificity of 60%.

- Disease-Free Survival Analysis

Kaplan-Meier -INTENSITY_BASED_TotalLesionGlycolysis

Case Processing Summary

INTENSITYBASED2	N total	N of events	Censored	
			N	Percentage
values below the cut-off point			5	33,3%
values greater than or equal to the cut-off point	21		5	23,8%
Global		26		27,8%

Survival table

INTENSITYBASED2		Time	Status	Cumulative proportion surviving over time		N of accumulated events	N of remaining cases
				Estimate	Standard error		
values below the point Cutting	1	2,467	0	.	.	0	
	2	12,233	1	,929	,069	1	
		15,633	1	,857	,094		
		16,633	1	,786	,110		
	5	17,667	1	,714	,121		
		17,733	1	,643	,128	5	
		19,800	1	,571	,132		
		20,533	1	,500	,134		
		21,900	1	,429	,132		
		22,667	1	,357	,128		5
		23,733	0	.	.		
		24,533	0	.	.		
		25,667	0	.	.		
		31,800	0	.	.		1
		44,900	1	,000	,000		0
values greater than or equal to to the cut-off point	1	4,433	1	,952	,046	1	
	2	9,700	1	,905	,064		
		10,433	1	,857	,076		
		10,500	1	,810	,086		
	5	10,633	1	,762	,093	5	
		10,767	1	,714	,099		
		11,567	1	,667	,103		
		12,033	1	,619	,106		
		12,700	1	,571	,108		
		14,267	1	,524	,109		
		14,367	1	,476	,109		
		15,167	1	,429	,108		
		15,267	1	,381	,106		
		16,633	1	,333	,103		
		22,433	1	,286	,099		
		23,100	0	.	.		5
		23,833	0	.	.		
		24,433	0	.	.		
		26,767	1	,190	,102		
		46,433	0	.	.		1
		55,000	0	.	.		0

Means and medians for survival time

INTENSITYBASED2	Mediaa				Medium			
			95% Confidence Interval				95% Confidence Interval	
	Estimate	Standard error	Lower limit	Upper limit	Estimate	Standard error	Lower limit	Upper limit
values below the carte point	27,807	3,657	20,639	34,975	20,533	1,964	16,683	24,383
values greater than or equal to the carte point	22,116	3,978	14,319	29,913	14,367	1,882	10,678	18,055
Global	24,827	3,012	18,924	30,731	17,667	2,464	12,837	22,496

a. The estimate is limited to the longest survival time, if censored.

Global comparisons

	Chi-square	gl	Sig.
**Log Rank (Mantel-Cox)**	**1,538**	**1**	**,215**

Test of equality of survival distributions for the different levels of INTENSITYBASED2.

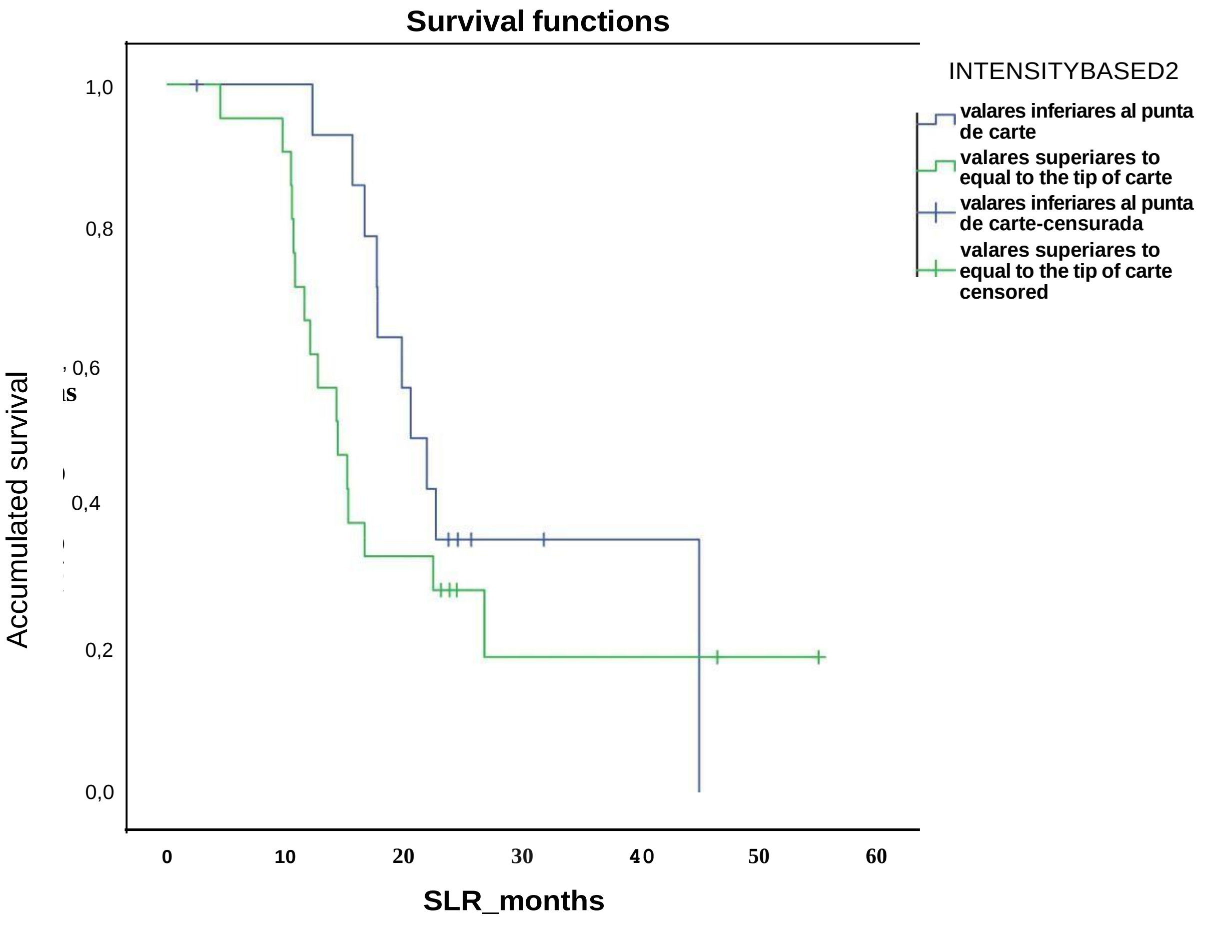

p=0.215 not statistically significant differences It seems that those with lower values are doing somewhat better.

Case Processing Summary

GLRLM2	N total	N of events	Censored	
			N	Percentage
values below the cut-off point				46,2%
values greater than or equal to the cut-off point				17,4%
Global		26		27,8%

Survival table

GLRLM2		Time	Status	Cumulative proportion surviving over time		N of accumulated events	N of remaining cases
				Estimate	Standard error		
values below the point	1	2,467	0	.	.	0	
Cutting	2	12,233	1	,917	,080	1	
		15,633	1	,833	,108		
		16,633	1	,750	,125		
	5	17,667	1	,667	,136		
		21,900	1	,583	,142	5	
		22,667	1	,500	,144		
		23,733	0	.	.		5
		24,533	0	.	.		
		25,667	0	.	.		
		31,800	0	.	.		
		44,900	1	,250	,191		1
		46,433	0	.	.		0
values greater than or equal to	1	4,433	1	,957	,043	1	
to the cut-off point	2	9,700	1	,913	,059		21
		10,433	1	,870	,070		
		10,500	1	,826	,079		
	5	10,633	1	,783	,086	5	
		10,767	1	,739	,092		
		11,567	1	,696	,096		
		12,033	1	,652	,099		
		12,700	1	,609	,102		
		14,267	1	,565	,103		
		14,367	1	,522	,104		
		15,167	1	,478	,104		
		15,267	1	,435	,103		
		16,633	1	,391	,102		
		17,733	1	,348	,099		
		19,800	1	,304	,096		
		20,533	1	,261	,092		
		22,433	1	,217	,086		5
		23,100	0	.	.		
		23,833	0	.	.		
	21	24,433	0	.	.		
		26,767	1	,109	,088		1
		55,000	0	.	.		0

Means and medians for survival time

GLRLM2	Mediaa				Medium			
	Estimate	Standard error	95% Confidence Interval		Estimate	Standard error	95% Confidence Interval	
			Lower limit	Upper limit			Lower limit	Upper limit
values below the carte point	31,728	4,099	23,693	39,762	22,667	9,959	3,146	42,187
values greater than or equal to the carte point	19,712	3,281	13,282	26,143	15,167	,799	13,602	16,732
Global	24,827	3,012	18,924	30,731	17,667	2,464	12,837	22,496

a. The estimate is limited to the longest survival time, if censored.

Global comparisons

	Chi-square	gl	Sig.
Log Rank (Mantel-Cox)	4,459	1	,035

Test for equality of survival distributions for different levels of GLRLM2.

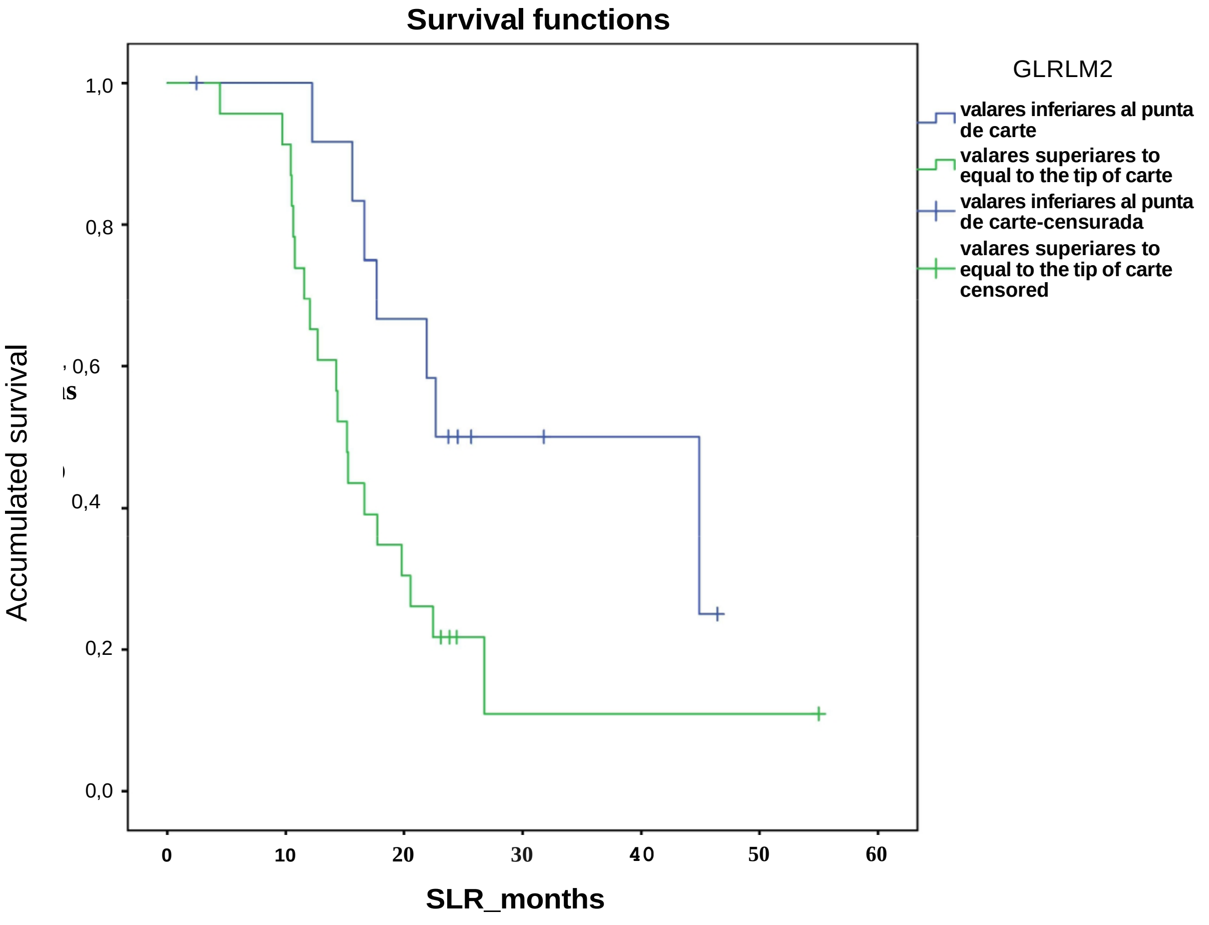

p=0.035 there are statistically significant differences in survival according to this variable. Let's look at the associated risk.

Variables in the equation

	B	SE	Wald	gl	Sig.	Exp(B)	95.0% CI for Exp(B)	
							Lower	Top
GLRLM2	-,912	,447	4,164	1	,041	,402	,167	,965

Means of covariates

	Media
GLRLM2	,343

p=0.041 and HR=Exp(B)=0.402 => Interpretation: patients with values below the cut-off point reduce the risk of relapse by 59.8% (1-HR) compared to those with values above or equal to the cut-off point.

Kaplan-Meier - GLSZM_ZoneSizeNonUniformity

Case Processing Summary

GLSZM2	N total	N of events	Censored	
			N	Percentage
values below the cut-off point				31,6%
values greater than or equal to the cut-off point				23,5%
Global		26		27,8%

Survival table

GLSZM2		Time	Status	Cumulative proportion surviving over time		N of accumulated events	N of remaining cases
				Estimate	Standard error		
values below the point	1	2,467	0	.	.	0	
Cutting	2	12,233	1	,944	,054	1	
		12,700	1	,889	,074		
		15,167	1	,833	,088		
	5	15,633	1	,778	,098		
		16,633	1	,722	,106	5	
		17,667	1	,667	,111		
		17,733	1	,611	,115		
		19,800	1	,556	,117		
		20,533	1	,500	,118		
		21,900	1	,444	,117		
		22,433	1	,389	,115		
		22,667	1	,333	,111		
		23,733	0	.	.		5
		24,433	0	.	.		
		24,533	0	.	.		
		25,667	0	.	.		
		31,800	0	.	.		1
		44,900	1	,000	,000		0
values greater than or equal to	1	4,433	1	,941	,057	1	
to the cut-off point	2	9,700	1	,882	,078		
		10,433	1	,824	,092		
		10,500	1	,765	,103		
	5	10,633	1	,706	,111	5	
		10,767	1	,647	,116		
		11,567	1	,588	,119		
		12,033	1	,529	,121		
		14,267	1	,471	,121		
		14,367	1	,412	,119		
		15,267	1	,353	,116		
		16,633	1	,294	,111		5
		23,100	0	.	.		
		23,833	0	.	.		
		26,767	1	,196	,109		
		46,433	0	.	.		1
		55,000	0	.	.		0

Means and medians for survival time

GLSZM2	Mediaa				Medium			
	Estimate	Standard error	95% Confidence Interval		Estimate	Standard error	95% Confidence Interval	
			Lower limit	Upper limit			Lower limit	Upper limit
values below the carte point	26,917	3,195	20,654	33,180	20,533	2,227	16,168	24,899
values greater than or equal to the carte point	21,679	4,417	13,022	30,336	14,267	1,921	10,502	18,031
Global	24,827	3,012	18,924	30,731	17,667	2,464	12,837	22,496

a. The estimate is limited to the longest survival time, if censored.

Global comparisons

	Chi-square	gl	Sig.
**Log Rank (Mantel-Cox)**	**1,602**	**1**	**,206**

Test for equality of survival distributions for different levels of GLSZM2.

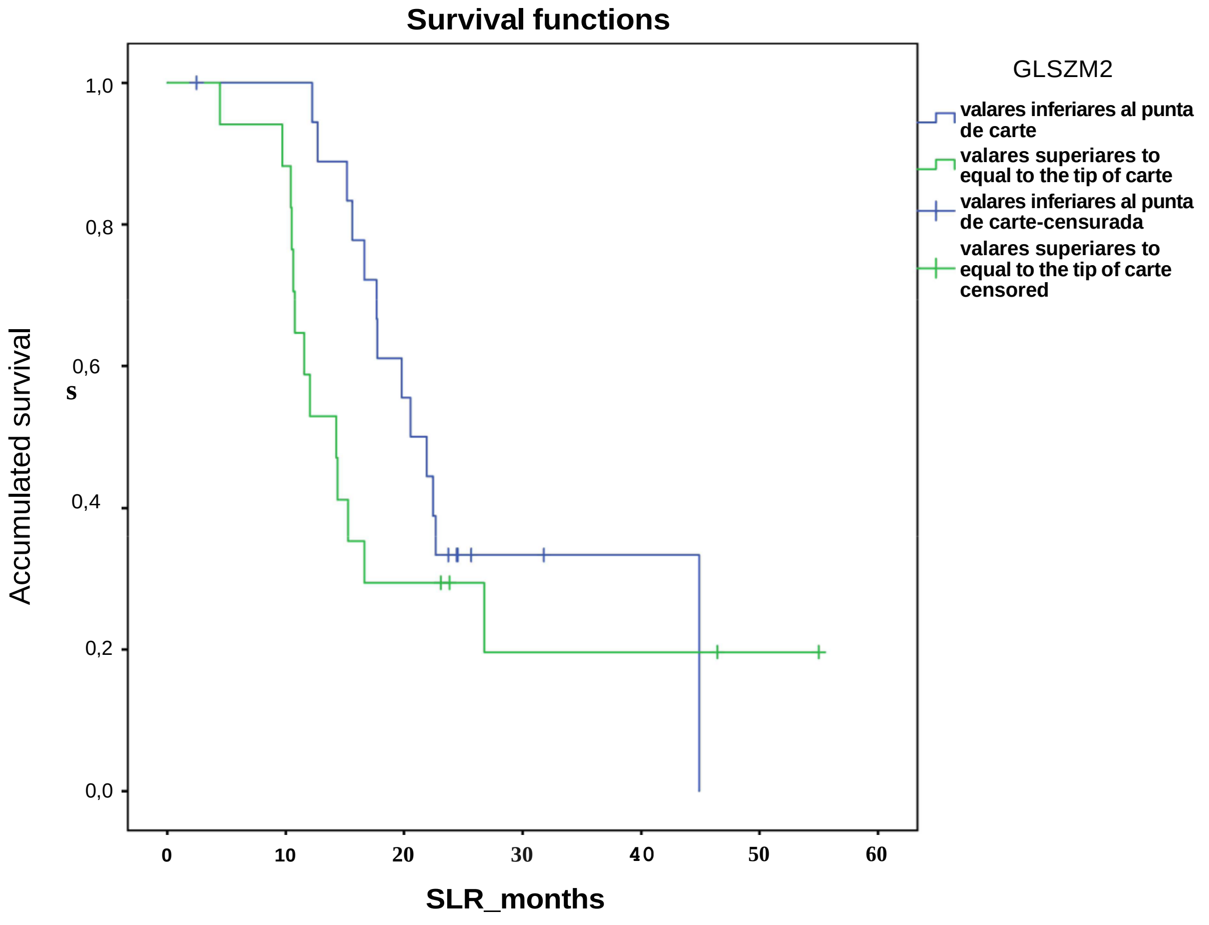

p=0.206 not statistically significant differences It seems that those with lower values are doing somewhat better.

- Cut-off points for INTENSITY_BASED_Kurtosis according to Exitus

COR Curve

Case Processing Summary

Exitus	N valid (per list)
Positive to Negative	21

Larger values of test result variables indicate a higher test for a positive true state.

a. The actual positive state is 1.

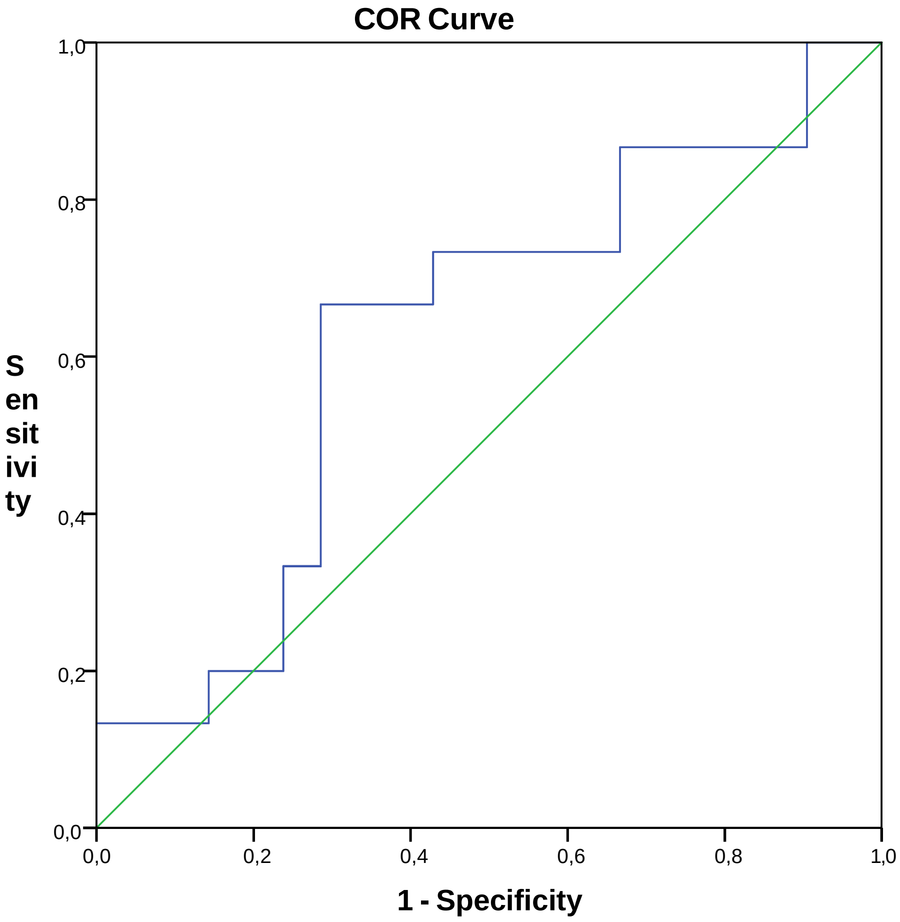

Area under the curve

Test result variables: INTENSITY-BASED_Kurtosis

Area	Standard error	Asymptotic significanceb	95% asymptotic confidence interval	
			Lower limit	Upper limit
,625	,097	,205	,435	,816

a. Under the non-parametric assumption

b. Null hypothesis: true area = 0.5

Curve coordinates

Test result variables: INTENSITY-B

Positive if it is greater than or equal to	Sensitivity	1 - Specificity
-1,83636433	1,000	1,000
-,741176292	1,000	,952
-,601902716	1,000	,905
-,557447048	,933	,905
-,513383338	,867	,905
-,441362713	,867	,857
-,388242385	,867	,810
-,303380309	,867	,762
-,233747088	,867	,714
-,196013723	,867	,667
-,148210785	,800	,667
-,072833230	,733	,667
,0341020789	,733	,619
,2283236567	,733	,571
,3920841118	,733	,524
,5164897229	,733	,476
,7166925360	,733	,429
1,009200321	,667	,429
1,308232742	,667	,381
1,474172776	,667	,333
1,859173642	,667	,286
2,349343230	,600	,286
2,997366088	,533	,286
3,517441503	,467	,286
3,733500682	,400	,286
4,146044082	,333	,286
4,392046682	,333	,238
4,574083976	,267	,238
4,874774887	,200	,238
5,588225956	,200	,190
6,633386465	,200	,143
8,316871033	,133	,143
9,989149585	,133	,095
10,71732210	,133	,048
13,09457176	,133	,000
36,93029581	,067	,000
59,66837615	,000	,000

a. The smallest cutoff value is the minimum observed test value minus 1 and the largest cutoff value is the maximum observed test value plus 1. All other cutoff values are the averages of the two consecutive requested observed test values.

There are two points that can be good:
Cut-off point 0.7166925360 which has a sensitivity of 73.3% and specificity of 57.1%.
Cut-off point 1.859173642 which has a sensitivity of 66.7% and specificity of 71.4%.

- Overall survival

Kaplan-Meier - INTENSITY_BASED_Kurtosis with the point 0.7166925360

Case Processing Summary

INTENSITYBASED_Kurtosis2	N total	N of events	Censored	
			N	Percentage
values below the cut-off point				75,0%
values greater than or equal to the cut-off point				45,0%
Global				58,3%

Survival table

INTENSITYBASED_Kurtosis2		Time	Status	Cumulative proportion surviving over time		N of events accumulated	Nof cases remaining
				Estimate	Standard error		
values below the point cutting	1	7,967	1	,938	,061	1	15
		17,633	1	,875	,083	2	14
		23,833	0	.	.	2	13
		24,533	0	.	.	2	12
	5	25,600	0	.	.	2	11
		25,667	0	.	.	2	10
		29,500	1	,788	,111	3	9
		30,867	0	.	.	3	8
		31,800	0	.	.	3	7
		36,033	0	.	.	3	6
		38,567	1	,656	,152	4	5
		40,667	0	.	.	4	4
		46,433	0	.	.	4	3
		48,000	0	.	.	4	2
		51,400	0	.	.	4	1
		85,767	0	.	.	4	0
values greater than or equal to the cut-off point	1	2,500	1	,950	,049	1	19
		13,333	1	,900	,067	2	18
		20,533	1	,850	,080	3	17
		20,600	1	,800	,089	4	16
	5	23,133	1	,750	,097	5	15
		23,733	0	.	.	5	14
		23,733	0	.	.	5	13
		24,433	0	.	.	5	12

Survival table

INTENSITYBASED_Kurtosis2	Time	Status	Cumulative proportion surviving over time		N of accumulated events	N of remaining cases
			Estimate	Standard error		
	26,300	1	,687	,107		
	29,133	1	,625	,114		
	35,067	1	,562	,119		
	45,067	1	,500	,121		
	45,633	1	,437	,121		
	49,167	0	.	.		
	51,067	0	.	.		5
	54,033	1	,350	,124		
	54,200	0	.	.		
	55,000	0	.	.		
	83,533	0	.	.		1
	92,433	0	.	.		0

Means and medians for survival time

INTENSITYBASED_Kurtosis2	Media[a]				Medium			
	Estimate	Standard error	95% confidence interval		Estimate	Standard error	95% confidence interval	
			Lower limit	Upper limit			Lower limit	Upper limit
values below the point cutting	65,528	8,552	48,766	82,289	.	.	.	.
values greater than or equal to the cut-off point	52,410	7,737	37,245	67,574	45,067	10,208	25,058	65,075
Global	57,917	6,412	45,350	70,484	54,033	7,655	39,029	69,038

a. The estimate is limited to the longest survival time, if censored.

Global comparisons

	Chi-square	gl	Sig.
Log Rank (Mantel-Cox)	1,373	1	,241

Test of equality of survival distributions for different levels of INTENSITYBASED_Kurtosis2.

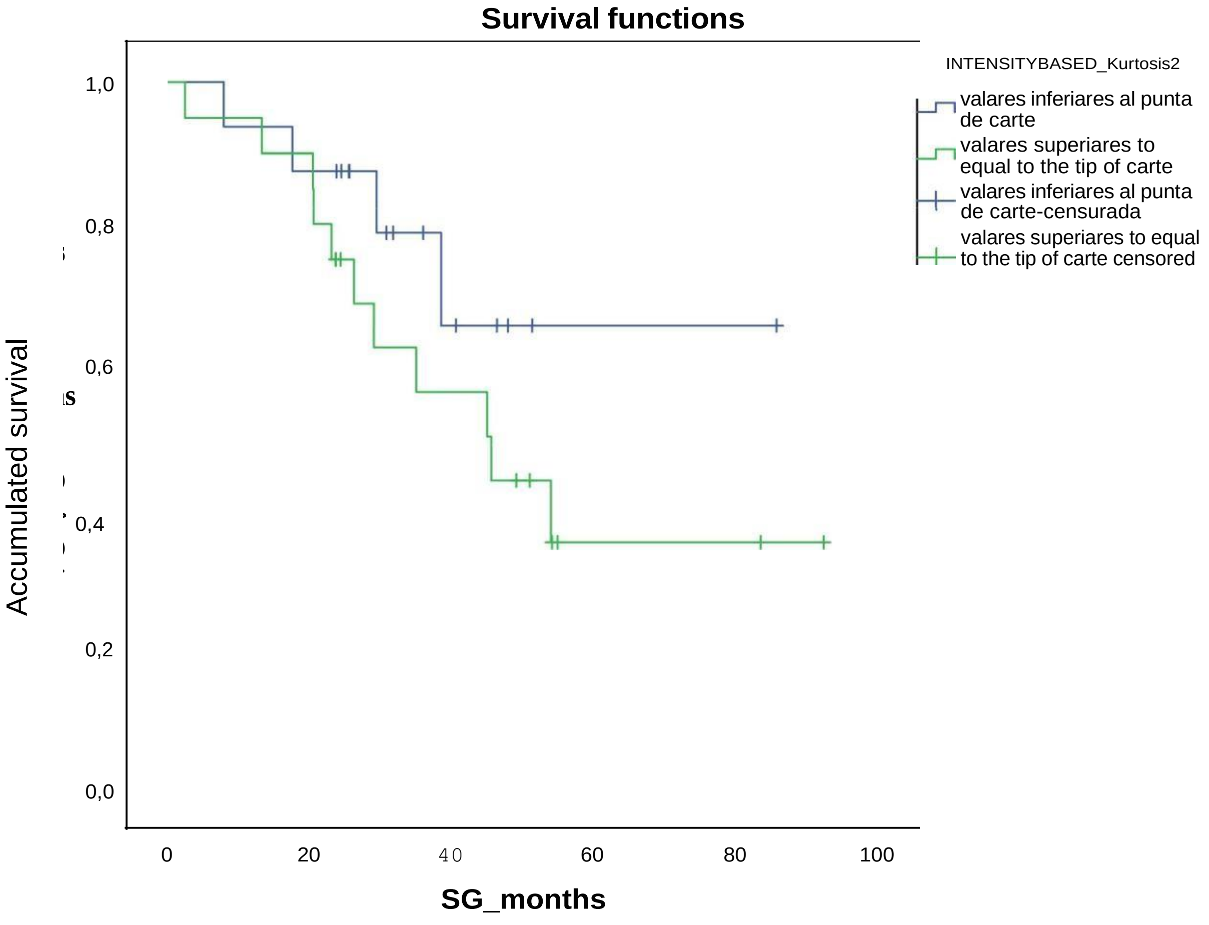

p=O. 241 no difference at the beginning the lines cross, it seems that from month 20 onwards the lower values are better.

Kaplan-Meier - INTENSITY_BASED_Kurtosis with point 1.85917364 2

Case Processing Summary

INTENSITYBASED _Kurtosis3	N total	N of events	Censored	
			N	Percentage
values below the carte point		5		75,0%
values greater than or equal to the carte point				37,5%
Global				58,3%

Survival table

INTENSITYBASED_Kurtosis3		Time	Status	Cumulative proportion surviving over time		N of accumulated events	N of remaining cases
				Estimate	Standard error		
values below the point	1	7,967	1	,950	,049	1	
Cutting	2	17,633	1	,900	,067		
		23,733	0	.	.		
		23,833	0	.	.		
	5	24,533	0	.	.		
		25,600	0	.	.		
		25,667	0	.	.		
		29,500	1	,831	,091		
		30,867	0	.	.		
		31,800	0	.	.		
		36,033	0	.	.		
		38,567	1	,738	,119		
		40,667	0	.	.		
		46,433	0	.	.		
		48,000	0	.	.		5
		51,400	0	.	.		
		54,033	1	,554	,183	5	
		54,200	0	.	.	5	
		85,767	0	.	.	5	1
		92,433	0	.	.	5	0
values greater than or equal to	1	2,500	1	,938	,061	1	
to the cut-off point	2	13,333	1	,875	,083		
		20,533	1	,813	,098		
		20,600	1	,750	,108		
	5	23,133	1	,688	,116	5	
		23,733	0	.	.	5	
		24,433	0	.	.	5	
		26,300	1	,611	,126		
		29,133	1	,535	,131		
		35,067	1	,458	,133		
		45,067	1	,382	,131		5
		45,633	1	,306	,125		
		49,167	0	.	.		
		51,067	0	.	.		
		55,000	0	.	.		1
		83,533	0	.	.		0

Means and medians for survival time

INTENSITYBASED_Kurtosis3	Media[a]				Medium			
			95% confidence interval				95% confidence interval	
	Estimate	Standard error	Lower limit	Upper limit	Estimate	Standard error	Lower limit	Upper limit
values below the point cutting	68,052	8,943	50,523	85,580	.	.	.	.
values greater than or equal to the cut-off point	44,372	7,402	29,864	58,880	35,067	10,876	13,750	56,383
Global	57,917	6,412	45,350	70,484	54,033	7,655	39,029	69,038

a. The estimate is limited to the longest survival time, if censored.

Global comparisons

	Chi-square	gl	Sig.
Log Rank (Mantel-Cox)	4,084	1	,053

Test of equality of survival distributions for different levels of INTENSITYBASED_Kurtosis3.

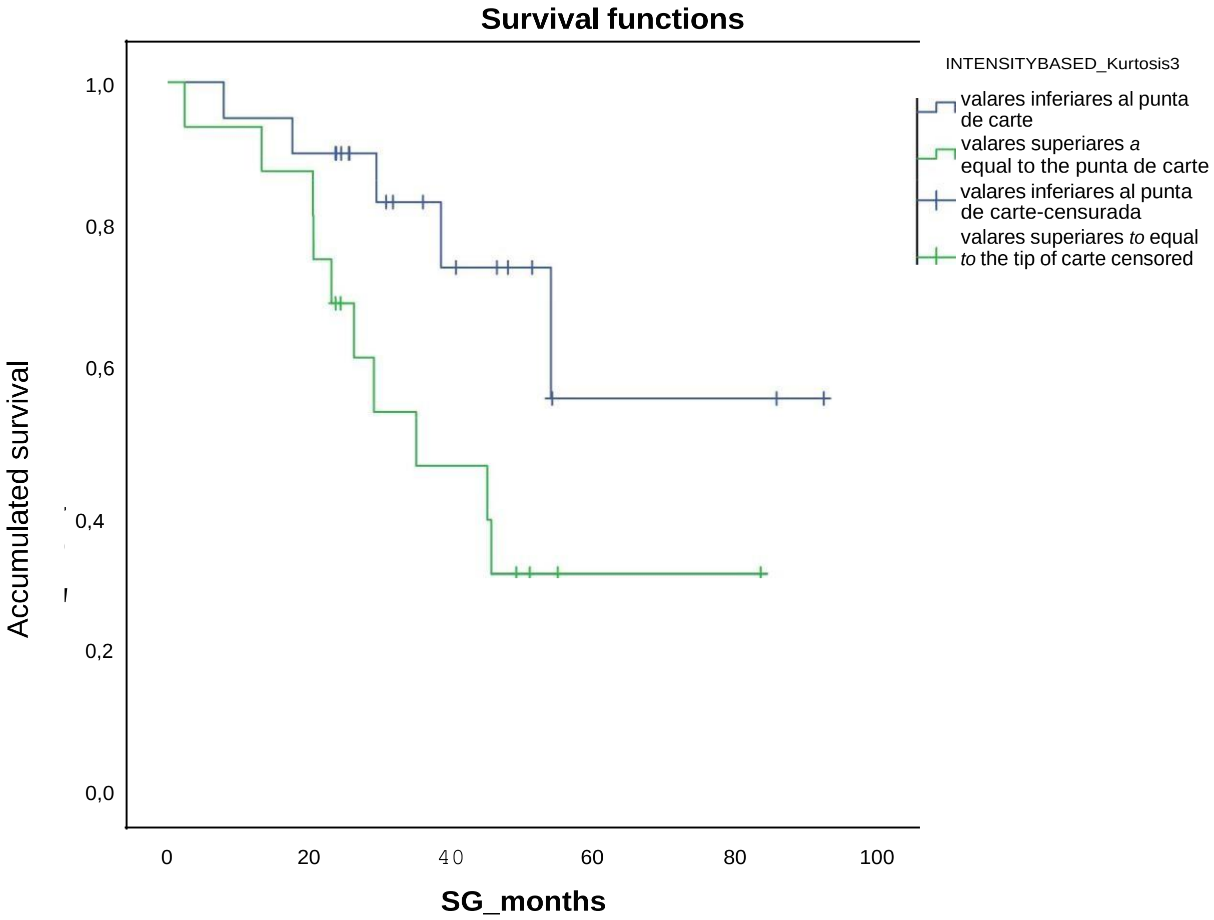

p=O. 053 differences are appreciated although the significance standards are not reached Patients with lower values seem to be doing better.

DeepL
Subscribe to DeepL Pro to translate larger documents.
Visit www.DeepL.com/pro for more information.

TYPE OF TREATMENT

Kaplan-Meier - Relapse-Free Survival

Case processing summary

Type of treatment	N total	N of events	Censored	
			N	Percentage
chemo + interval surgery	20	15	5	25,0%
initial surgery + chemo	8	5	3	37,5%
chemo alone	8	6	2	25,0%
Global	36	26	10	27,8%

Survival table

Type of treatment		Time	State	Cumulative proportion surviving over time		N of accumulated events	N of remaining cases
				Estimate	Standard error		
chemo + interval surgery	1	9,700	1	,950	,049	1	19
	2	10,433	1	,900	,067	2	18
	3	10,500	1	,850	,080	3	17
	4	12,033	1	,800	,089	4	16
	5	12,700	1	,750	,097	5	15
	6	15,167	1	,700	,102	6	14
	7	15,633	1	,650	,107	7	13
	8	16,633	1	,600	,110	8	12
	9	17,667	1	,550	,111	9	11
	10	19,800	1	,500	,112	10	10
	11	20,533	1	,450	,111	11	9
	12	21,900	1	,400	,110	12	8
	13	22,433	1	,350	,107	13	7
	14	22,667	1	,300	,102	14	6
	15	23,733	0	.	.	14	5
	16	23,833	0	.	.	14	4
	17	24,433	0	.	.	14	3
	18	31,800	0	.	.	14	2
	19	44,900	1	,150	,118	15	1
	20	55,000	0	.	.	15	0
initial surgery + chemo	1	10,633	1	,875	,117	1	7
	2	14,367	1	,750	,153	2	6
	3	16,633	1	,625	,171	3	5
	4	17,733	1	,500	,177	4	4
	5	24,533	0	.	.	4	3
	6	25,667	0	.	.	4	2
	7	26,767	1	,250	,198	5	1
	8	46,433	0	.	.	5	0
chemo alone	1	2,467	0	.	.	0	7

2	4,433	1	,857	,132	1	6
3	10,767	1	,714	,171	2	5

Survival table

Type of treatment	Time	State	Cumulative proportion surviving over time		N of accumulated events	N of remaining cases
			Estimate	Standard error		
4	11,567	1	,571	,187	3	4
5	12,233	1	,429	,187	4	3
6	14,267	1	,286	,171	5	2
7	15,267	1	,143	,132	6	1
8	23,100	0	.	.	6	0

Medians and medians for survival time

Type of treatment	Media[a]				Median			
			95% confidence interval				95% confidence interval	
	Estimate	Standard error	Lower limit	Upper limit	Estimate	Standard error	Lower limit	Upper limit
chemo + interval surgery	26,375	3,711	19,101	33,649	19,800	3,205	13,518	26,082
initial surgery + chemo	25,721	5,229	15,472	35,970	17,733	4,777	8,371	27,096
chemo alone	13,090	1,968	9,234	16,947	12,233	,873	10,523	13,944
Global	24,827	3,012	18,924	30,731	17,667	2,464	12,837	22,496

a. The estimate is limited to the longest survival time, if censored.

Global comparisons

	Chi-square	gl	Sig.
Log Rank (Mantel-Cox)	4,895	2	,095

Test for equality of survival distributions for different levels of Treatment type.

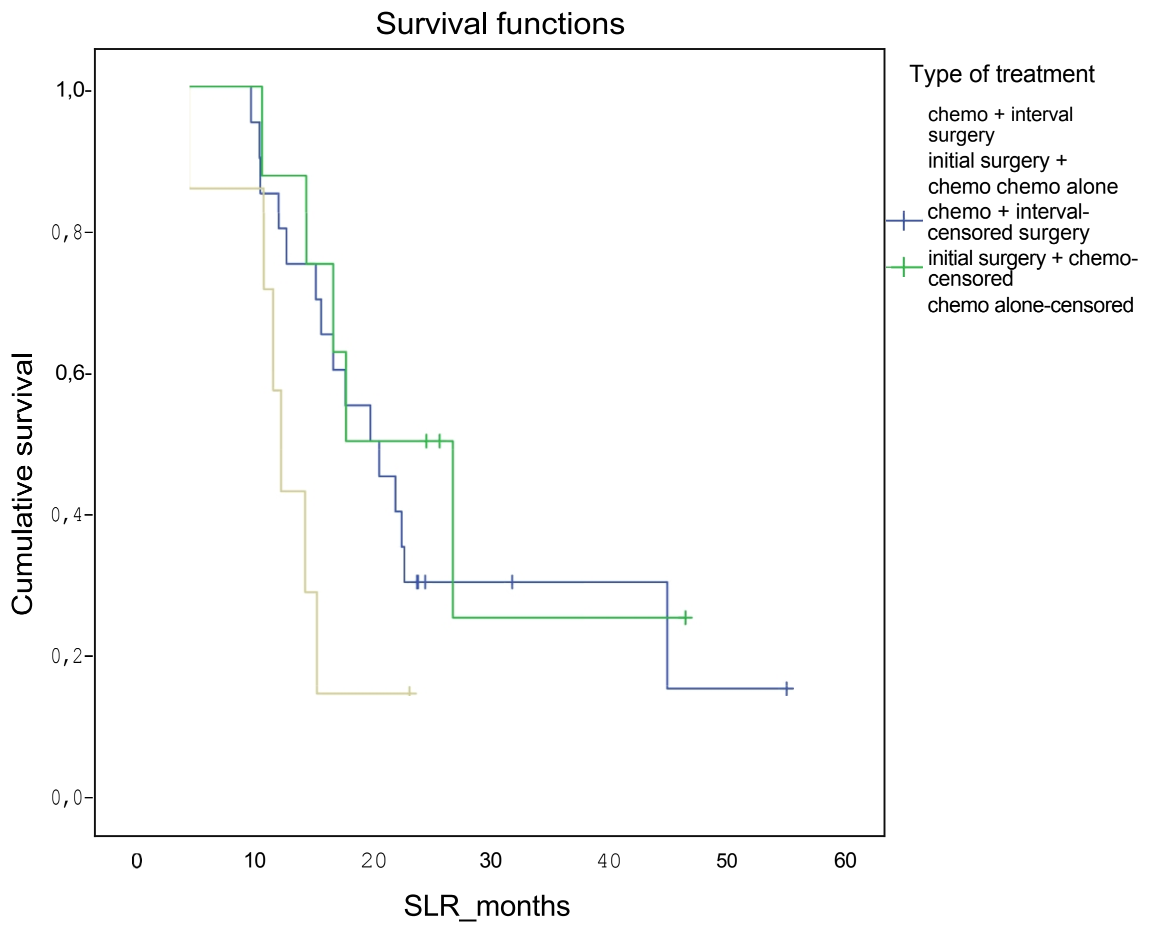

p=0.095, some differences can be seen although the significance standards are not reached

Kaplan-Meier - Global Survival

Case processing summary

Type of treatment	N total	N of events	Censored	
			N	Percentage
chemo + surgery for interval	20	9	11	55,0%
initial surgery + chemo	8	1	7	87,5%
chemo alone	8	5	3	37,5%
Global	36	15	21	58,3%

Survival table

Type of treatment		Time	State	Cumulative proportion surviving over time: Estimate	Cumulative proportion surviving over time: Standard error	N of accumulated events	N of remaining cases
chemo + surgery for interval	1	17,633	1	,950	,049	1	19
	2	20,533	1	,900	,067	2	18
	3	23,733	0	.	.	2	17
	4	23,733	0	.	.	2	16
	5	23,833	0	.	.	2	15
	6	24,433	0	.	.	2	14
	7	29,133	1	,836	,088	3	13
	8	29,500	1	,771	,102	4	12
	9	30,867	0	.	.	4	11
	10	31,800	0	.	.	4	10
	11	35,067	1	,694	,117	5	9
	12	38,567	1	,617	,127	6	8
	13	45,067	1	,540	,133	7	7
	14	45,633	1	,463	,134	8	6
	15	48,000	0	.	.	8	5
	16	51,067	0	.	.	8	4
	17	54,033	1	,347	,142	9	3
	18	54,200	0	.	.	9	2
	19	55,000	0	.	.	9	1
	20	92,433	0	.	.	9	0
initial surgery + chemo	1	24,533	0	.	.	0	7
	2	25,667	0	.	.	0	6
	3	26,300	1	,833	,152	1	5
	4	46,433	0	.	.	1	4
	5	49,167	0	.	.	1	3
	6	51,400	0	.	.	1	2
	7	83,533	0	.	.	1	1
	8	85,767	0	.	.	1	0
chemo alone	1	2,500	1	,875	,117	1	7
	2	7,967	1	,750	,153	2	6
	3	13,333	1	,625	,171	3	5
	4	20,600	1	,500	,177	4	4
	5	23,133	1	,375	,171	5	3
	6	25,600	0	.	.	5	2
	7	36,033	0	.	.	5	1
	8	40,667	0	.	.	5	0

Medians and medians for survival time

Type of treatment	Media[a]				Median			
			95% confidence interval				95% confidence interval	
	Estimate	Standard error	Lower limit	Upper limit	Estimate	Standard error	Lower limit	Upper limit
chemo + interval surgery	56,695	7,593	41,813	71,576	45,633	7,690	30,560	60,706
initial surgery + chemo	75,856	9,048	58,122	93,589	.	.	.	.
chemo alone	23,692	5,120	13,656	33,728	20,600	6,930	7,018	34,182
Global	57,917	6,412	45,350	70,484	54,033	7,655	39,029	69,038

a. The estimate is limited to the longest survival time, if censored.

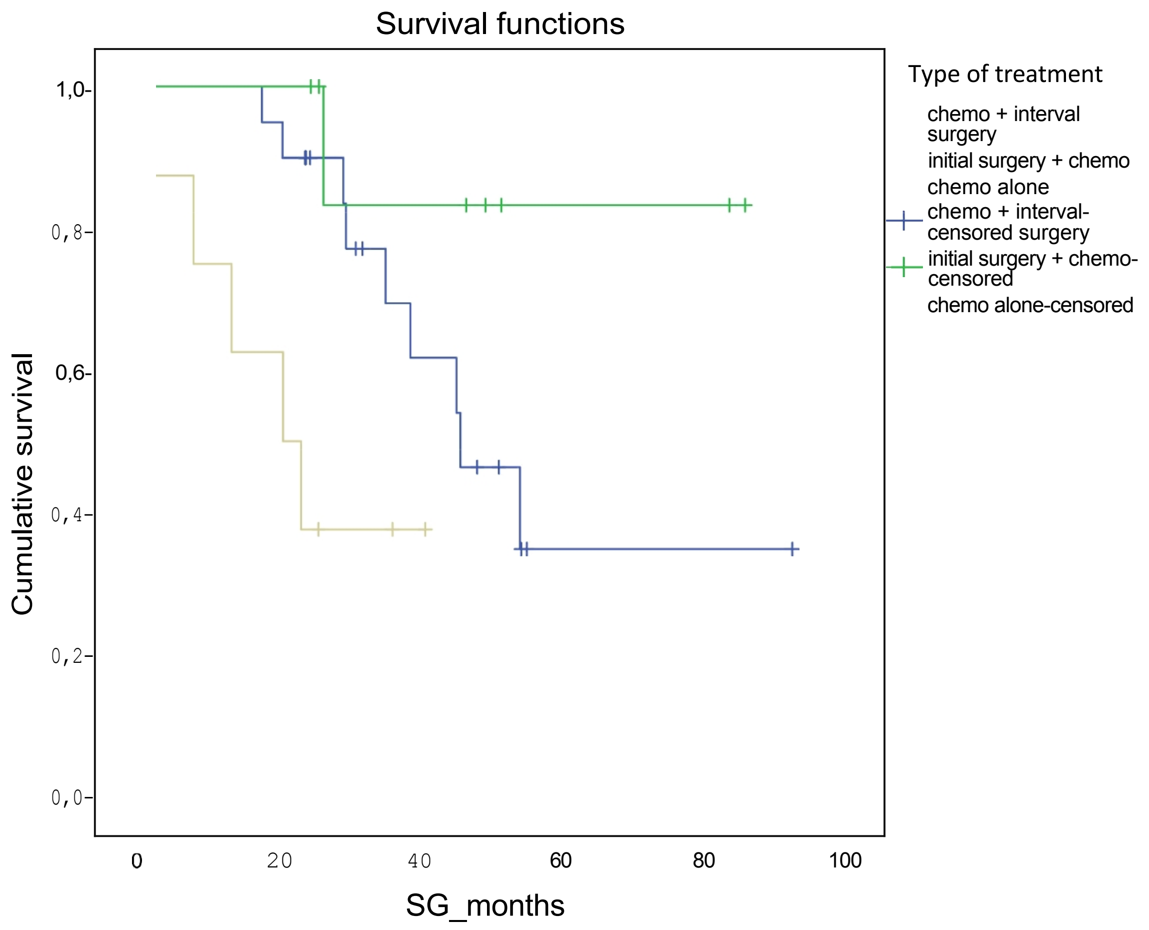

No p-value due to lack of events.

TREATMENT CHEMO VS CHEMO+SURGERY

We will combine chemo + interval surgery and chemo + initial surgery for comparison with chemo alone.

Kaplan-Meier - Relapse-free Survival

Case processing summary

ChemoSurgery	N total	N of events	Censored	
			N	Percentage
chemo alone	8	6	2	25,0%
chemo + surgery	28	20	8	28,6%
Global	36	26	10	27,8%

Survival table

ChemoSurgery		Time	State	Cumulative proportion surviving over time		N of accumulated events	N of remaining cases
				Estimate	Standard error		
chemo alone	1	2,467	0	.	.	0	7
	2	4,433	1	,857	,132	1	6
	3	10,767	1	,714	,171	2	5
	4	11,567	1	,571	,187	3	4
	5	12,233	1	,429	,187	4	3
	6	14,267	1	,286	,171	5	2
	7	15,267	1	,143	,132	6	1
	8	23,100	0	.	.	6	0
chemo + surgery	1	9,700	1	,964	,035	1	27
	2	10,433	1	,929	,049	2	26
	3	10,500	1	,893	,058	3	25
	4	10,633	1	,857	,066	4	24
	5	12,033	1	,821	,072	5	23
	6	12,700	1	,786	,078	6	22
	7	14,367	1	,750	,082	7	21
	8	15,167	1	,714	,085	8	20
	9	15,633	1	,679	,088	9	19
	10	16,633	1	.	.	10	18
	11	16,633	1	,607	,092	11	17
	12	17,667	1	,571	,094	12	16
	13	17,733	1	,536	,094	13	15
	14	19,800	1	,500	,094	14	14
	15	20,533	1	,464	,094	15	13
	16	21,900	1	,429	,094	16	12
	17	22,433	1	,393	,092	17	11
	18	22,667	1	,357	,091	18	10
	19	23,733	0	.	.	18	9
	20	23,833	0	.	.	18	8
	21	24,433	0	.	.	18	7
	22	24,533	0	.	.	18	6
	23	25,667	0	.	.	18	5
	24	26,767	1	,286	,097	19	4
	25	31,800	0	.	.	19	3
	26	44,900	1	,190	,101	20	2
	27	46,433	0	.	.	20	1
	28	55,000	0	.	.	20	0

Medians and medians for survival time

ChemoSurgery	Media"				Median			
	Estimate	Standard error	95% confidence interval		Estimate	Standard error	95% confidence interval	
			Lower limit	Upper limit			Lower limit	Upper limit
chemo alone	13,090	1,968	9,234	16,947	12,233	,873	10,523	13,944
chemo + surgery	26,920	3,3ñi9	20,337	33,ñi04	19,800	2,800	14,312	2ñi,288
Global	24,827	3,012	18,924	30,731	17,667	2,464	12,837	22,496

a. The estimate is limited to the longest survival time, if censored.

Global comparisons

	Chi-square	gl	Sig.
Log Rank (Mantel-CoX)	4,596	1	,052

Test for equality of survival distributions for different levels of ChemoSurgery.

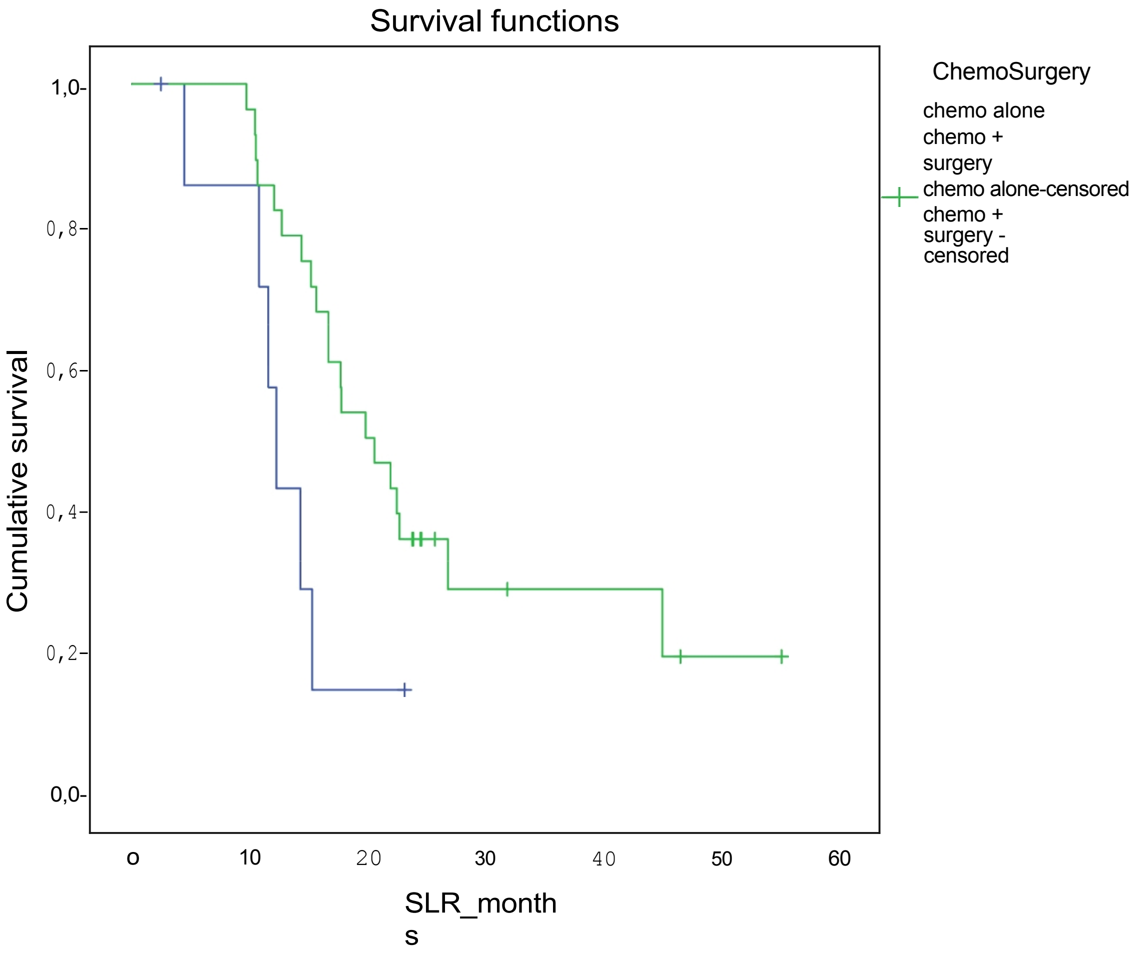

p=0.052 is shown
some difference in survival according to Chemo or Chemo+Surgery although the standards of significance are not reached This may be

due to the sample size we have.

Kaplan-Meier - Global Survival

Case processing summary

ChemoSurgery	N total	N of events	Censored	
			N	Percentage
chemo alone	8	5	3	37,5%
chemo + surgery	28	10	18	64,3%
Global	36	15	21	58,3%

Survival table

ChemoSurgery		Time	Status	Cumulative proportion surviving over time		N of events accumulated	N of remaining cases
					Estimate	Standard error	
chemo alone	1	2,500	1				
	2	7,967	1	,875	,117	1	7
	3	13,333	1	,750	,153	2	6
	4	20,600	1	,625	,171	3	5
	5	23,133	1	,500	,177	4	4
	6	25,600	0	,375	,171	5	3
	7	36,033	0	.	.	5	2
	8	40,667	0	.	.	5	1
chemo + surgery	1	17,633	1	.	.	5	0
	2	20,533	1	,964	,035	1	27
	3	23,733	0	,929	,049	2	26
	4	23,733	0	.	.	2	25
	5	23,833	0	.	.	2	24
	6	24,433	0	.	.	2	23
	7	24,533	0	.	.	2	22
	8	25,667	0	.	.	2	21
	9	26,300	1	.	.	2	20
	10	29,133	1	,882	,065	3	19
	11	29,500	1	,836	,076	4	18
	12	30,867	0	,789	,085	5	17
	13	31,800	0	.	.	5	16
	14	35,067	1	.	.	5	15
	15	38,567	1	,737	,094	6	14
	16	45,067	1	,684	,101	7	13
	17	45,633	1	,631	,106	8	12
	18	46,433	0	,579	,110	9	11
	19	48,000	0	.	.	9	10
	20	49,167	0	.	.	9	9
	21	51,067	0	.	.	9	8
	22	51,400	0	.	.	9	7
	23	54,033	1	.	.	9	6
	24	54,200	0	,482	,127	10	5
	25	55,000	0	.	.	10	4
				.	.	10	3

Survival table

ChemoSurgery	Time	State	Cumulative proportion surviving over time		N of accumulated events	N of remaining cases
			Estimate	Standard error		
26	83,533	0	.	.	10	2
27	85,767	0	.	.	10	1
28	92,433	0	.	.	10	0

Medians and medians for survival time

ChemoSurgery	Mediaa				Median			
	Estimate	Standard error	95% confidence interval		Estimate	Standard error	95% confidence interval	
			Lower limit	Upper limit			Lower limit	Upper limit
chemo alone	23,692	5,120	13,656	33,728	20,600	6,930	7,018	34,182
chemo + surgery	63,750	6,713	50,593	76,908	54,033	.	.	.
Global	57,917	6,412	45,350	70,484	54,033	7,655	39,029	69,038

a. The estimate is limited to the longest survival time, if censored.

Global comparisons

	Chi-square	gl	Sig.
Log Rank (Mantel-Cox)	7,834	1	,005

Test for equality of survival distributions for different levels of ChemoSurgery.

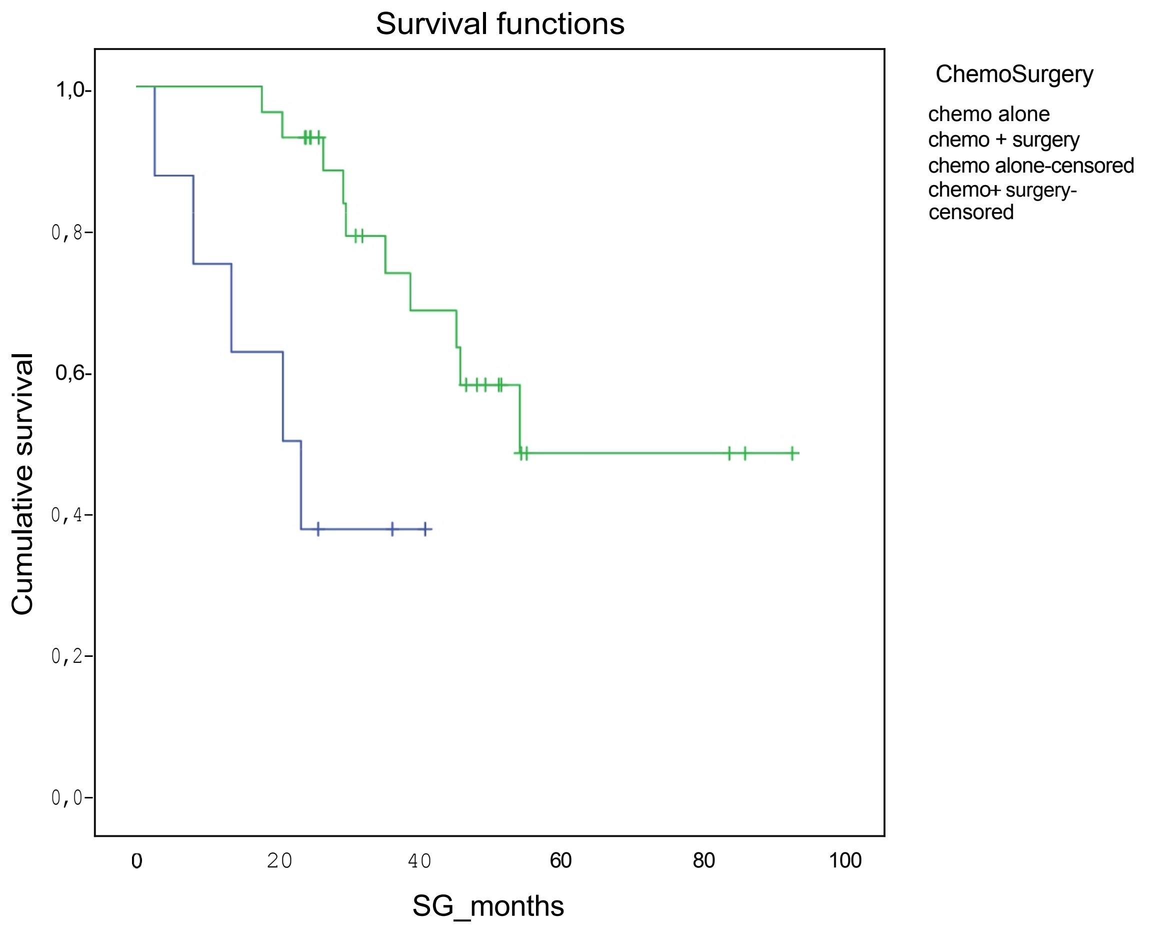

p=0.005 difference in survival according to Chemo or Chemo+Surgery. Let's see what is the risk associated with this variable.

Cox regression

Variables in the equation

	B	SE	Wald	gl	Sig.	Exp(B)	95.0º / CI for Exp(B)	
							Inferior	Superior
ChemoSurgery	-1,526	,596	6,548	1	,011	,217	,068	,700

Means of covariates

	Media
ChemoSurgery	,778

p=0.011 HR=0.217 95%CI (0.968, 0.700) => presenting chemo+surgery reduces the risk of relapse by 78.3% (1-HR) in comparison to receiving chemo

alone.

MULTIVARIATE MODEL

1) RELAPSE

Cox regression - categorical GLRLM (cutoff point) with type of treatment.

Variables in the equation

	B	SE	Wald	gl	Sig.	Exp(B)	95.0% CI for Exp(B)	
							Inferior	Superior
GLRLM2	,858	,453	3,585	1	,048	2,359	1,070	5,735
Type of treatment	,240	,263	,830	1	,362	1,271	,759	2,129

Means of covariates

	Media
GLRLM2	,657
Type of treatment	1,629

```
p-value of GLRLM_RLNU p=0.048 has changed but maintains significance.
HR=2.359 CI95%=(1.070, 5.735) => Adjusting for treatment time,
presenting values greater than or equal to the cutoff point increases
2.359 times the
 risk of relapse compared to having lower values.
```

Cox regression - GLRLM continuous

Variables in the equation

	B	SE	Wald	gl	Sig.	Exp(B)	95.0% CI for Exp(B)	
							Inferior	Superior
GLRLM_RunLengthNonUniformity	,000	,000	2,426	1	,119	1,000	1,000	1,000
Type of treatment	,192	,273	,495	1	,482	1,211	,710	2,066

Means of covariates

	Media
GLRLM_RunLengthNonUniformity	11216,084
Type of treatment	1,629

GLRLM p-value p=0.119 not significant.

Cox regression - Taking as variable treatment chemo vs. chemo+surgery categorical GLRLM

Variables in the equation

	B	SE	Wald	gl	Sig.	Exp(B)	95.0% CI for Exp(B)	
							Inferior	Superior
GLRLM2	,778	,462	2,835	1	,052	2,178	,980	5,388
ChemoSurgery	-,741	,496	2,230	1	,135	,476	,180	1,261

Means of covariates

	Media
GLRLM2	,657
ChemoSurgery	,800

p-value of GLRLM_RLNU p=0.052 has changed and we lose significance. Differences are seen although the significance standards are not reached.

Cox regression - Taking as variable treatment chemo vs chemo+surgery GLRLM continuous GLRLM

Variables in the equation

	B	SE	Wald	gl	Sig.	Exp(B)	95.0% CI for Exp(B)	
							Inferior	Superior
GLRLM_RunLengthNonUniformity	,000	,000	1,121	1	,209	1,000	1,000	1,000
ChemoSurgery	-,683	,569	1,440	1	,230	,505	,166	1,541

Means of covariates

	Media
GLRLM_RunLengthNonUniformity	11216,084
ChemoSurgery	,800

GLRLM p-value p=0.209 not significant.

Cox regression - categorical GLSZM (cutoff point) with type of treatment

Variables in the equation

	B	SE	Wald	gl	Sig.	Exp(B)	95.0% CI for Exp(B)	
							Inferior	Superior
GLSZM2	,338	,468	,524	1	,469	1,403	,561	3,507
Type of treatment	,217	,313	,480	1	,489	1,242	,673	2,293

Means of covariates

	Media
GLSZM2	,486
Type of treatment	1,629

p-value of GLSZM p=0.469 not significant.

Cox regression - continuous GLSZM

Variables in the equation

	B	SE	Wald	gl	Sig.	Exp(B)	95.0% CI for Exp(B)	
							Inferior	Superior
GLSZM_ZoneSizeNonUniformity	,000	,000	3,129	1	,057	1,000	1,000	1,001
Type of treatment	,154	,293	,278	1	,598	1,167	,657	2,072

Means of covariates

	Media
GLSZM_ZoneSizeNonUniformity	1301,301
Type of treatment	1,629

p-value of GLSZM p=0.057 differences are appreciated although the significance standards are not reached.

Cox regression - Taking as variable treatment chemo vs. chemo+surgery GLRLM categorical GLRLM

Variables in the equation

	B	SE	Wald	gl	Sig.	Exp(B)	95.0% CI for Exp(B)	
							Inferior	Superior
GLSZM2	,231	,452	,261	1	,609	1,260	,520	3,055
ChemoSurgery	-,863	,549	2,470	1	,116	,422	,144	1,238

Means of covariates

	Media
GLSZM2	,486
ChemoSurgery	,800

p-value of GLSZM p=0.609 not significant.

Cox regression - Taking as variable treatment chemo vs chemo+surgery GLRLM continuous GLRLM

Variables in the equation

	B	SE	Wald	gl	Sig.	Exp(B)	95.0% CI for Exp(B)	
							Inferior	Superior
GLSZM_ZoneSizeNonUniformity	,000	,000	2,791	1	,095	1,000	1,000	1,001
ChemoSurgery	-,752	,499	2,271	1	,132	,471	,177	1,254

Means of covariates

	Media
GLSZM_ZoneSizeNonUniformity	1301,301
ChemoSurgery	,800

p-value of GLSZM p=0.095 not significant.

2) EXITUS

Cox regression - Categorical kurtosis (cutoff point) with type of treatment

Variables in the equation

	B	SE	Wald	gl	Sig.	Exp(B)	95.0% CI for Exp(B)	
							Inferior	Superior
INTENSITYBASED_Kurtosis3	,268	,398	,453	1	,501	1,307	,599	2,849
Type of treatment	,339	,263	1,658	1	,198	1,404	,838	2,352

Means of covariates

	Media
INTENSITYBASED_Kurtosis3	,429
Type of treatment	1,629

p-value of Kurtosis p=0.501 not significant.

Cox regression - Continuous kurtosis

Variables in the equation

	B	SE	Wald	gl	Sig.	Exp(B)	95.0% CI for Exp(B)	
							Inferior	Superior
INTENSITY-BASED_Kurtosis	,020	,019	1,175	1	,108	1,021	,984	1,059
Type of treatment	,316	,269	1,389	1	,239	1,372	,811	2,323

Means of covariates

	Media
INTENSITY-BASED_Kurtosis	4,291
Type of treatment	1,629

p-value of Kurtosis p=0.108 not significant.

Cox regression - Taking as variable treatment chemo vs chemo + surgery - Cutoff Kurtosis

Variables in the equation

	B	SE	Wald	gl	Sig.	Exp(B)	95.0% CI for Exp(B)	
							Inferior	Superior
INTENSITYBASED_Kurtosis3	,155	,402	,149	1	,700	1,168	,531	2,569
ChemoSurgery	-,968	,490	3,908	1	,058	,380	,145	1,092

Means of covariates

	Media
INTENSITYBASED_Kurtosis3	,429
ChemoSurgery	,800

p-value of Kurtosis p=0.700 not significant.

Cox regression - Taking as variable treatment chemo vs chemo+surgery Continuous Kurtosis

Variables in the equation

	B	SE	Wald	gl	Sig.	Exp(B)	95.0% CI for Exp(B)	
							Inferior	Superior
INTENSITY-BASED_Kurtosis	,015	,019	,630	1	,428	1,015	,978	1,053
ChemoSurgery	-,943	,501	3,547	1	,060	,389	,146	1,039

Means of covariates

	Media
INTENSITY-BASED_Kurtosis	4,291
ChemoSurgery	,800

p-value of Kurtosis p=0.428 not significant.
